# Brewers’ Spent Grain from Different Types of Malt: A Comprehensive Evaluation of Appearance, Structure, Chemical Composition, Antimicrobial Activity, and Volatile Emissions

**DOI:** 10.3390/molecules30132809

**Published:** 2025-06-30

**Authors:** Aleksander Hejna, Joanna Aniśko-Michalak, Katarzyna Skórczewska, Mateusz Barczewski, Paweł Sulima, Jerzy Andrzej Przyborowski, Hubert Cieśliński, Mariusz Marć

**Affiliations:** 1 Institute of Materials Technology, Poznan University of Technology, Piotrowo 3, 61-138 Poznan, Poland; joanna.anisko@put.poznan.pl (J.A.-M.); mateusz.barczewski@put.poznan.pl (M.B.); 2 Department of Polymer Technology, Gdańsk University of Technology, Narutowicza 11/12, 80-233 Gdansk, Poland; 3 Faculty of Chemical Technology and Engineering, Bydgoszcz University of Science and Technology, Seminaryjna 3 Street, 85-326 Bydgoszcz, Poland; katarzyna.skorczewska@pbs.edu.pl; 4 Department of Genetics, Plant Breeding and Bioresource Engineering, University of Warmia and Mazury in Olsztyn, Plac Łódzki 3, 10-724 Olsztyn, Poland; pawel.sulima@uwm.edu.pl (P.S.); jerzy.przyborowski@uwm.edu.pl (J.A.P.); 5 Department of Molecular Biotechnology and Microbiology, Gdańsk University of Technology, Narutowicza 11/12, 80-233 Gdansk, Poland; hcieslin@pg.edu.pl; 6 Department of Analytical Chemistry, Gdańsk University of Technology, Narutowicza 11/12, 80-233 Gdansk, Poland; mariusz.marc@pg.edu.pl

**Keywords:** brewing industry, brewers’ spent grain, waste management, chemical composition, antioxidant properties

## Abstract

Beer is the third most popular beverage in the world, and its production is distributed uniformly between the biggest continents. Considering the environmental aspects, the utilization of brewing by-products, mainly brewers’ spent grain (BSG), is essential on a global scale. The beer revolution, lasting over a few decades, significantly diversified the beer market in terms of styles, and therefore, also its by-products, which should be characterized appropriately prior to further application. Herein, the presented study investigated the unprecedented number of 22 different variants of brewers’ spent grain, yielded from the production of various beer styles, enabling their proper comparison. A comprehensive by-product characterization revealed an almost linear relationship (Pearson correlation coefficients exceeding 0.9) between the color parameters (L*, a*, browning index) of beer and generated spent grain, enabling a prediction of BSG appearance based on beer color. Applying wheat or rye malts increased the content of extractives by over 40%, reducing cellulose content by as much as 45%. Thermal treatments of malts (kilning or smoking) also reduced extractive content and limited antioxidant activity, often by over 30%. A lack of husk for wheat or rye reduced the crystallinity index of spent grain by 21–41%, while the roasting of barley efficiently decomposed the less stable compounds and maintained the cellulose crystalline structure. All the analyzed BSG samples were characterized by low volatile emissions and very limited antimicrobial activity. Therefore, their harmfulness to human health and the environment is limited, broadening their potential application range.

## 1. Introduction

Light lager is by far the most popular beer style globally, due to its central position in the brewing concerns’ offerings [1,2]. However, as a result of the beer revolution, which has lasted over the last decades in the USA and Europe, the current beer market offers a multiplicity of beer choices unprecedented in previous years [3]. Microbreweries and craft breweries began to offer various beer styles regularly that were previously often only available locally, in the region of their origin [4]. A whole new variety of styles, from less to more bitter and from lighter to darker, also required substantial changes in the production schemes [5]. Different styles require different raw materials, primarily malts, differing in the malting procedure, e.g., the kilning and curing times or temperature, resulting in varying extracts, color, or protein content. During mashing, they provide different features to the wort, affecting its color, aroma, and flavor. Except for the most conventional pilsener and pale ale barley malts, other varieties became more popular [6]. Breweries currently use malts made from barley, wheat, or rye, which are caramelized, roasted, or even smoked. As a result, the composition and structure of the generated by-product, the brewers’ spent grain (BSG), is also different for various beer styles. In the beginning, when only very small breweries were producing different, less popular beer styles, the issue of by-product heterogeneity was considered negligible. However, the enormous success of the craft beer revolution induced changes in the whole beer market, which resulted in a broadening of the beer palettes offered by big brewing concerns [5]. Therefore, the production scale of beers other than light lagers has significantly increased over the last few years, affecting the generation of by-products.

Over the last few years, global beer production has varied between 1.91 and 1.97 billion hectoliters annually [7]. The share of craft breweries in the beer market differs locally. In the U.S. beer market, over 13% of beer volume originates from craft breweries [8]. In European countries, the craft beer revolution started later and has been developing more slowly due to a consolidated beer culture and the multiplicity of local microbreweries focused on traditional, local beer styles [5]. Nevertheless, the share of craft beer in the whole market constantly increases, enhancing the diversity of available beer styles and generated BSG. It accounts for ~85% of total beer manufacturing by-products [9], which, considering production volume, yields almost 12 million tons of BSG annually [10].

So far, only a few works have investigated the different types of BSG. Naibaho and Korzeniowska [11] aimed to identify the differences in BSG from different breweries. They collected by-products from eight different breweries in Poland, Germany, and Estonia. Insights on their chemical composition and physical properties have been provided. However, no information has been provided regarding applied malts, their composition, or at least the style and parameters of the beer from which particular BSG samples originated. A similar approach has been presented in the work of Robertson et al. [12], who analyzed BSG from ten commercial breweries from the European Union (EU) and South Africa. The only information on BSG’s origin was that it was a residue from either lager or ale production.

More details have been provided in the works of Jin et al. [13] and Pereira de Freitas et al. [14]. The first one [13] reported the details of the chemical composition of four BSG types obtained from different craft breweries. Two types included only barley malt; one was supplemented with oat and chocolate malt, and the last one included barley malt, spelt, and rice hulls. Insights on fiber, starch, protein, lipid, ash, amino acid, phenolic, and mineral contents have been provided. Nevertheless, the lack of more details about the content of applied malts limits the conclusions that can be drawn from the study. Accordingly, the second work [14] investigated the composition of BSG from India pale ale (solely Pale Ale barley malt), Baltic porter (Vienna/Munich malts with cocoa nibs), and Weissbier (light barley malt and wheat malt in a 1:1 ratio). Despite providing details on the chemical composition, the first two BSG types originated from commercial breweries, while the third one was from homebrewing, which notably affects the mashing process and its efficiency. Similar to the previous works, no details on the content of particular malts have been provided.

Nevertheless, advancement in the efficient management of brewing by-products requires a more comprehensive analysis of precisely determined BSG samples, which could be further transferred to industry-originated materials. Herein, in the presented study, we investigated twenty-two variants of BSG mirroring the potential recipes applied for manufacturing various beers from light lagers, through wheat and rye beers, as well as pale, amber, and brown ales, to stouts or porters, including even the application of smoked and peated malts, which also have their followers.

## 2. Results and Discussion

### 2.1. Visual Assessment of the Obtained BSG

**Figure 1:**
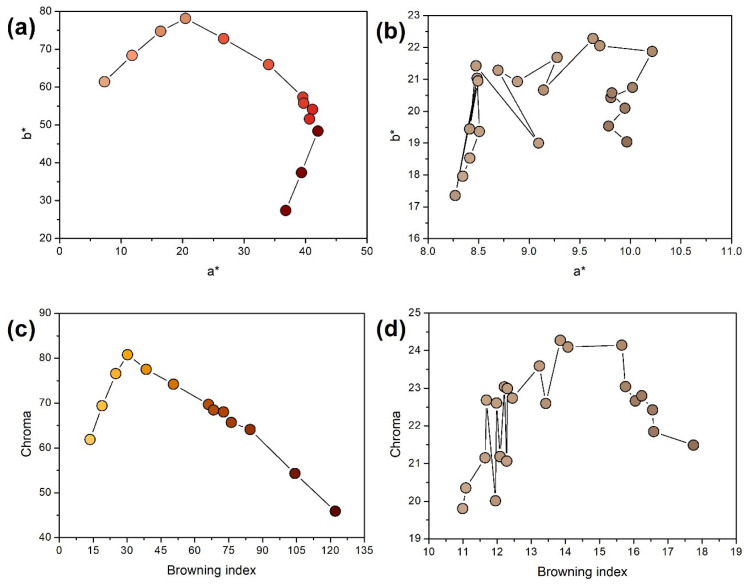
The relationship between the a* and b* parameters, and the chroma and browning index, of (**a**,**c**) beer, and the (**b**,**d**) brewers’ spent grain samples.

Table 1 provides details on the appearance of the analyzed BSG samples and their comparison with the appearance of the produced beer, which has been directly affected by the applied malts. Considering the differences in beer color, significant variations have been noted, but these were limited in the case of the BSG samples. It was attributed to the substantial impact of malt color on the beer’s appearance [15]. The values of the beers’ BI aligned with the color parameters, as well as with the introduction of malts kilned at higher temperatures. Such an effect has been attributed to the generation of melanoidins during malts’ manufacturing, whose content could be indirectly assessed by the determination of the browning index (BI) [16]. According to Pepa et al. [17], the complex nature of non-enzymatic browning (including Maillard reactions yielding melanoidins) includes three phases, as presented in Figure 1. The first phase can be associated with the increase of the b* parameter and the yellowing of the material. Then, a significant turn of the color changes toward the red zone was noted and expressed by the a* increase with a slight reduction of the b* values. Finally, the most intense browning was associated with a significant darkening and shifting toward the initial achromatic zone. Due to the considerable differences between the colors of particular beers, all of these phases can be easily seen in Figure 1a. At the same time, the flattening of color changes for the BSG affected the course of the color changes (Figure 1b). Moreover, in the case of BSG, the differences in particle size showed a significant impact on color determination, which has been repeatedly reported in the literature [18,19].

Color parameters have also been used to determine chroma and hue. Hue is one of the main parameters distinguished by color theory and is represented quantitatively by a single number corresponding to an angular position around a central or neutral point or axis on a color space coordinate diagram. The determination of hue for fillers is related to the frequent use of this parameter in the identification of biomass [20], e.g., for the determination of the analysis of green-colored plant parts, as is performed for the content of chlorophylls in leaves [21]. Chroma determines the color saturation and is associated with its intensity; however, despite the increasing BI and deepening brown color of beer, the descending trend of chroma has been noted (Figure 1c,d). The assessment of the browning of natural products, including various types of food, has been repeatedly reported as very challenging due to the complex nature of the process, and includes color darkening, except for the hue changes [22,23]. Therefore, chroma could only be applied as a browning indicator to some extent. Pepa et al. [17] suggested that chroma may represent the browning phenomenon up to L* values of ~70, which aligns with the presented results. At the same time, BI has been reported as an efficient browning indicator up to L* values of ~10.

Considering the BSG color, the span of the obtained values was limited, but the trend was similar to that of beer color. Very high Pearson correlation coefficients (PCCs) between the L* and a* parameters, as well as the BI of beer and BSG, have been noted, with values of 0.94, 0.91, and 0.94, respectively. It clearly indicated that although malts’ characteristics significantly contribute to the appearance of the beer, their whole coloring potential was not transferred to the wort during mashing. Moreover, based on the presented data, the color of the generated BSG could be quite precisely assessed by knowing the characteristics of the beer produced. Such a phenomenon should be considered very beneficial for the potential utilization of BSG because the by-product from brewing various beer styles could be dedicated to the particular product and its desired appearance.

### 2.2. Composition and Chemical Structure of the Obtained BSG

Table 2 presents the composition of the obtained BSG samples, and their antioxidant activity determined by the 2,2-diphenyl-1-picrylhydrazyl (DPPH) assay. The cold water extractives (CWEs) included extraneous components, e.g., inorganic compounds, tannins, gums, sugars, and coloring matter, while the hot water extractives (HWEs) also included starches [24]. These values significantly depended on the grain type and malting procedure. The wheat and rye malts showed higher numbers of CWEs and HWEs due to the lower content of structural components—cellulose, hemicellulose, and lignin [25]. When considering the barley malts, their kilning enhanced their extractive content due to the breakdown of cell-wall constituents, but too high of a temperature probably caused their degradation, limiting their CWE and HWE contents.

For comparison, Table 3 summarizes the literature reports on the contents of the most commonly reported components of BSG. Notably, the majority of cited studies [11,26,27,28,29,30,31,32,33] have not provided any details on the composition of the analyzed BSG. Three of them [34,35,36] reported that the BSG was obtained from barley malts. Two of them indicated a combination of barley with corn (8:2 ratio) [37] or with rice (6:4 ratio) [38]. One work indicated the type of beer, a traditional pilsner [39], which may suggest solely using pilsener barley malt in combination with non-malt components. Only Jin et al. [13] provided more detailed information about the four analyzed BSG types. Two of them were composed only of barley malt; one was a combination of barley malt with oat and chocolate malt, and the last one was a combination of barley malt with spelt and rice hulls. Nevertheless, no details about the contents of the particular components have been provided.

It can be seen that the values obtained in the presented study were relatively in line with the literature data. The differences may arise from two different aspects. The first one was related to the differences in the particular protocols applied for the determination of dietary fiber and its specific components: cellulose, hemicellulose, and lignin [40,41]. Recently, Fahey et al. [42] postulated that different laboratories should conduct comprehensive, collaborative studies to evaluate new methods of modification for the existing ones to enhance the precision of dietary fiber determination. Another aspect implicating the differences between the presented research and the literature data is associated with the composition of the analyzed BSG samples. As mentioned above, most of the studies dealt with the most conventional BSG, which probably originated from the brewing of a light lager, so it was composed either solely of Pilsen malt or its combination with non-malt components like corn or rice. Considering the overwhelming popularity of light lagers over other beer styles [43], as well as the similarity between particular studies, it can be assumed that the majority of studies that have not reported on the composition of BSG also dealt with by-products from light lager production.

In the presented study, multiple BSG types originated not only from conventional Pilsen malt but also from various special barley malts (caramel, chocolate, dark, and smoked), as well as rye and wheat (regular wheat malt and Grodziski). As a result, the contents of the neutral detergent fiber (NDF) and proteins for particular BSG samples were noticeably lower than those reported for conventional barley BSG types. At the same time, similar values have been reported by Jin et al. [13] for BSGs obtained from mashing barley, spelt, and rice hulls, 24%_DW_ of NDF and 15.2%_DW_ of proteins, which was even less than in the presented study. A relatively low NDF content (35.4%_DW_) has also been noted for BSG, consisting of barley malt, oat malt, and chocolate malt. These values have been attributed to the characteristics of craft breweries, including low brewhouse efficiency, high grain and malt input, coarse milling, and limited sparging compared to large brewing companies. On the other hand, they could be associated with the use of non-conventional raw materials, especially spelt, whose grain does not contain the hull typical of barley, limiting the NDF content [44].

Except for the abovementioned components, the BSG contained noticeable amounts of the phytochemicals responsible for its antioxidant activity, which has been investigated by many researchers [30,45,46]. The literature reports even indicated that BSG contains up to 2 wt% of phenolics, which are characterized by potent antioxidant activity [47,48]. Unfortunately, due to the multiplicity of applied extraction methods and antioxidant assays, the reported activity significantly differed.

The DPPH assay indicated a relatively low value of Trolox equivalent antioxidant capacity (TEAC), which could be associated with the form of phenolics in BSG, as well as the applied drying procedure. According to Kittibunchakul et al. [49], conventional hot-air drying significantly affected the phenolic content and antioxidant activity of plant-based materials. Therefore, higher antioxidant activity has been reported for freeze-dried BSG [37]. Moreover, in plant-based materials, phenolic compounds exist in a free or bound form. The share of bound phenolics in BSG is noticeably higher (almost tripled) than that of the free form, which may affect the obtained results [50]. Bound phenolics are covalently linked to plant material and cannot be extracted by simple water or aqueous/organic solvent mixtures [51]. To assess the content of bound phenolics, extraction should be accompanied by the application of microwaves, alkaline, or enzymatic hydrolysis [52,53,54].

The application of microwave-assisted extraction by Moreira et al. [52] resulted in ~15 times higher antiradical power compared to the presented study, while ~5 times higher values have been reported by Kitrytė et al. [55] for supercritical carbon dioxide extraction. Studies using conventional solvent extraction reported similar values to those of the presented study [45,56].

Table 2 also points to the differences in antioxidant activity of particular BSG samples originating from different styles of beer. These results aligned with the antioxidant potential analysis of the varying beer types. The most significant variation has been noted for the application of wheat malt, which confirms the data reported by Fogarasi et al. [57], who analyzed the antioxidant activity of eight different types of wheat and the resulting malts and compared them to the common 4-row barley. For both grains and malts, barley was characterized by ~5 times higher DPPH scavenging activity. This effect was not observed for the application of Grodziski malt (also wheat malt), which may be attributed to the presence of additional phenolic compounds induced by malt smoking [58]. The application of rye malt, on the other hand, noticeably increased BSG antioxidant potential. According to Shopska et al. [59], rye malt showed around 30% higher DPPH scavenging activity than Pilsen malt and this was 186% higher when compared to wheat malt.

Petrón et al. [56] indicated the reduction of BSG antioxidant potential with an increasing share of dark Cara20 and Cara120 malts. Özcan et al. [60] pointed to the decrease in phenolic content during elongated kilning, which can be an issue, e.g., in the case of Special B malt. Tomková-Drábková et al. [61] suggested that kilning temperatures exceeding 65 °C reduce antioxidant potential due to the degradation of phenolic acids. On the other hand, the roasting of malts can facilitate the release of phenolics [62]. However, Granato et al. [63] analyzed the content of phenolics and the antioxidant activity of 29 beers (18 lagers and 11 brown ales), which directly indicated a correlation between the analyzed features and beer color resulting from the application of caramel or dark malts. Similar findings have been reported by Gąsior et al. [64]. Socha et al. [65] analyzed 11 different dark beers from Germany, the Czech Republic, and Poland. Despite variations between the particular samples, the phenolics’ content was significantly higher than that reported for light beers [66]. Liguori et al. [67] reported a 6% increase in beer antioxidant activity and a 28% rise in polyphenols content resulting from the substitution of 15 wt% of pale ale malt with Caraamber. Similar results have been reported by Koren et al. [68], Piazzon et al. [69], and Habschied et al. [70]. Considering the reported data, it has been noted that the antioxidant benefits resulting from the malting procedure of darker malts were transferred to the beers, pointing to the high efficiency of the mashing procedure. The results may suggest that these malting procedures also facilitated the release of compounds initially present in barley in a bound form.

### 2.3. Fourier Transform Infrared Spectroscopy and X-Ray Diffraction Analysis of the Obtained BSG

Fourier transform infrared spectroscopy (FTIR), and X-ray diffraction (XRD) analyses have been performed to assess the differences in the crystalline structure between particular BSG samples. The diffractograms and spectra are presented in Figures S1 and S2 in the Supplementary Data. Their appearance was similar to that reported for BSG by other researchers [71,72]. The XRD patterns indicated the presence of the cellulose crystalline phase at (002) on 2θ = 22–23° with broad diffraction in the range of 30–55°, pointing to the amorphous components. They pointed to the presence of cellulose type I [73]. Considering the FTIR spectra, they all showed the presence of bands characteristic of the stretching vibrations of hydroxyl groups (~3300 cm^−1^), C-H bonds (2930 and 2850 cm^−1^), the carbonyls present in lignin or proteins (1740 and 1636 cm^−1^), the C-N bonds in amine groups (1248 cm^−1^), and multiple C-O bonds present in the structure of lignocellulose materials (1150 and 1017 cm^−1^) [74,75].

Due to the complex nature of the analyzed samples and the relatively similar contents of the particular components (cellulose, hemicellulose, lignin) possessing similar structures and chemical moieties, the FTIR spectra revealed hardly any differences. A deeper investigation was conducted, and the values of the total crystallinity index (TCI), lateral order index (LOI), and hydrogen bonding index (HBI) were calculated, which are provided in Table S3 along with the cellulose crystallinity index (CCI) values calculated from the XRD results. It can be seen that the CCI was significantly reduced for the application of the wheat and rye malts, which has been attributed to the different grain structures and lack of husk, which was mainly composed of cellulose [76]. Moreover, noticeable CCI decreases have been noted for the BSG samples originating from the malts subjected to elongated kilning, like Special B or the Smoked malts. Similar effects have been reported for barley by Skendi et al. [77] and by Lekjing and Venkatachalam [78] for rice malts.

Considering the FTIR results, due to the aforementioned similarities in spectra, no straightforward information could be extracted, which was expressed by the low PCC values (provided in Tables S4 and S5) calculated for the relationships between the content of particular BSG components, the color parameters, and the CCI, TCI, LOI, or HBI values. The only strong correlation was observed between cellulose content and the CCI calculated from the XRD results (PCCs of 0.885 and 0.800, respectively, for all the samples after excluding the BSG samples containing wheat or rye malts).

**Figure 2:**
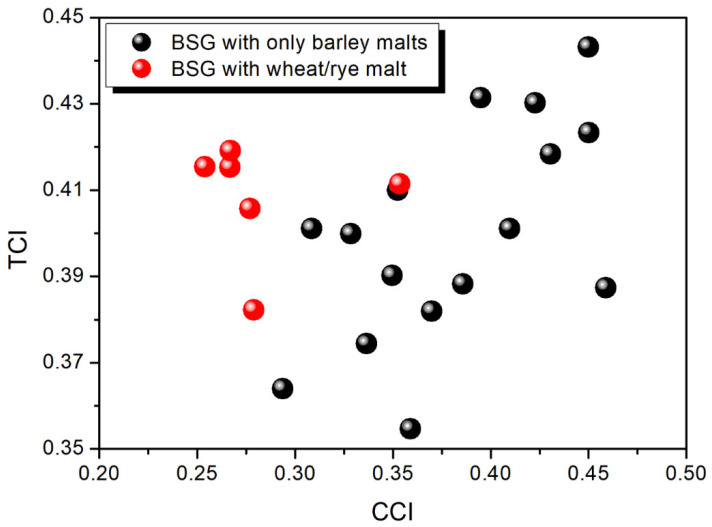
The relationship between the total crystallinity index and the cellulose crystallinity index calculated for the analyzed BSG samples.

Figure 2 presents the values of the CCI calculated from the XRD results and a comparison with the TCI determined based on the results of the FTIR analysis. The peak height method was applied for the CCI assessment, which, despite its affordability and simplicity, showed some drawbacks, making it a comparative method rather than one used to determine absolute values [79]. Nevertheless, the purpose of the presented study was to compare different BSG samples, so this method was selected. The comparison of the CCI and TCI values revealed hardly any proportionality. However, this changed after the exclusion of the BSG samples containing wheat or rye malts, which were characterized by different compositions (the PCC value shifted from 0.237 to 0.561). Such an effect could be attributed to the limitations of the FTIR technique itself, reported previously [80], as well as to the significant differences associated with the structure of grains.

### 2.4. Assessment of BSG Antimicrobial Activity

As mentioned above, food, agricultural, and other plant-based wastes contain a significant portion of phytochemicals. These compounds often possess not only antioxidant but also antimicrobial activity [45,81]. Depending on the further application of BSG, antimicrobial activity may be desired, e.g., to enhance the stability of the material or protect it against microorganisms. Therefore, its assessment should be considered a vital issue, providing important insights into further management.

Table 4 presents the results of testing the antibacterial activity of selected spent grains using the disk diffusion test. Based on the presented results, due to the absence of “clear zones” around the agar disk containing various BSG types, it may be concluded that none of the tested samples showed antibacterial activity against the tested bacterial strains.

Notably, instead of clear zones in the case of some tested BSG samples, a “zone of more intense bacterial growth” (ZMIBG) of the tested bacterial strains appeared around the analyzed agar-BSG disks (90 of 110 tested samples). This phenomenon was observed mainly for the tested gram-positive bacterial strains: *S. aureus, S. epidermidis,* and *St. pneumoniae*. In the case of the gram-negative bacteria *E. coli* and *P. aeruginosa*, this phenomenon was observed less frequently. For *P. aeruginosa*, the presence of ZMIBGs was rarely observed (only for several types of the examined BSG—see Table 4). In contrast, for this species of bacteria, increased yellow–green dye production by *P. aeruginosa* was often observed around agar disks containing the tested BSG (16 of 22 tested samples) in comparison to the control agar disk without BSG. The presence of pigments in “intensive growth zones” was also noted for all the agar–BSG disks tested on an LB-agar inoculated with the *St. pneumoniae* strain KBMiM.

In our opinion, the “intensive growth zones” phenomenon cannot be explained only by the diffusion of “pigments”, present in the analyzed BSG, into the agar medium. This is due to the fact that no changes in the color and transparency of the LA medium, on which no bacteria were inoculated, were observed around the agar disks placed on sterile LA agar plates and incubated under the same conditions as the Petri plates used in the disk diffusion assay. Moreover, analogous results were observed while testing the potential antibacterial activity of deep eutectic solvents using the disk diffusion assay [82]. In that study, the largest ZMIBGs were observed around the paper disks soaked with deep eutectic solvents containing glucose and choline chloride and placed on an LB-agar medium, inoculated with the *P. aeruginosa* strain ATCC 27853 (Figure 3 in the cited paper [82]). It seems possible that the diffusion of glucose (the preferred carbon source for bacterial growth) and choline chloride (an additional carbon and nitrogen source) from the paper disk to an LB-agar may create conditions for the faster growth of the bacterial cells in the vicinity of the disk, which was probably the reason for the observation of the ZMIBGs. Therefore, in the presented study, the ZMIBGs observed, especially around the agar disks, containing particular BSG samples may also result from the diffusion of chemical compounds from the BSG to the LB-agar that can enrich the growth medium with additional carbon and nitrogen sources in the vicinity of agar disks, especially considering the relatively high protein content of the analyzed samples (Table 2).

The presented results have not directly investigated the changes in the composition and properties of the BSG samples yielded from a subjection to various microorganisms. However, the lack of inhibition zones indicated that the included phytochemicals and melanoidins, which contributed to antioxidant performance, did not show antimicrobial activity. Such a phenomenon provided positive views on the potential biodegradability of BSG-containing materials, to be potentially developed in the future, like polymer-based composites or engineered wood materials. It should be considered an essential insight into future engineering and the development of such waste-based materials.

### 2.5. Volatile Organic Compound Emissions from BSG

The assessment of volatile organic compound (VOC) emissions should be considered essential during works on waste and biomass valorization. These materials, previously subjected to various processes, may be partially degraded or decomposed, which may yield unfavorable emissions. Moreover, in their primary applications, they may be used only as single components of final products. Still, recycling methods often aim to increase the share of waste in developed materials, which can amplify the impact of emissions on human health and the environment. Table 5 provides details on the VOCs detected during the analysis.

It can be seen that several aromatic hydrocarbons have been detected among the VOCs, which could be mainly attributed to their abundance in the environment, induced by the commonness of petroleum-based materials in everyday life [83]. Even storing BSG samples in plastic containers could induce such emissions, especially considering their low magnitude [84]. The limit of detection (L_D_) or limit of quantification (L_Q_) was not exceeded for most of the analyzed samples. However, the increasing temperature of kilning slightly increased the emissions of aromatics, which can be particularly seen for Roasted Barley. Such an effect could be attributed to the decomposition of lignocellulose material during malt kilning [85]. Moreover, the smoking of malt yielded increased aromatic emissions, which have been repeatedly reported for other smoked food products [86,87]. An increase was particularly noted for smoked and peated malts, while limited emissions were observed for Grodziski malt. Such an effect could be attributed to the differences in the grain structure and the type of wood used. According to Hitzel et al. [88], smoking meat with oak reduced aromatic emissions by ~30% compared to beech wood.

Moreover, due to the plant origin of BSG and our previous results [89], the emissions of terpenes and terpenoids have been analyzed. They have often been detected in different plants or even neat cellulose since they are the most prominent family of phytochemicals (over 80,000), representing around 60% of known natural products [90,91]. For the analyzed samples, the most abundant were pinenes, camphene, limonene, and menthol, partially aligning with the literature reports [92]. Considering the type of grain, no straightforward impact on terpene and terpenoid emissions has been noted. However, the smoking of malts with beech wood noticeably increased the amount of detected (+/−)-β-citronellol, α-cedrene, α-humulene, and (+)-cedrol, which aligned with the results of van Meeningen et al. [93], who reported noticeably higher terpene emissions for beech than for oak wood.

Moreover, Table S6 provides information on the potential threats caused by the detected compounds according to the two internationally accepted systems, NFPA 704: the Standard System for the Identification of the Hazards of Materials for Emergency Response maintained by the U.S.-based National Fire Protection Association and the Globally Harmonized System of Classification and Labelling of Chemicals (GHS) [94,95]. Many detected compounds were categorized as threats, which could be related to their potential toxicity and low flash point, which increased the possibility of ignition. Nevertheless, the aforementioned low magnitude of emissions significantly limited the posed threats. Moreover, most of the detected compounds, especially terpenes and terpenoids, can hardly be treated as volatile. However, there is no actual value of vapor pressure, which clearly determines if a compound is considered a VOC; the United States Environmental Protection Agency has exempted solvents in consumer products with a maximum vapor pressure of 0.1 mm Hg (~13 Pa) at 20 °C [96]. Keeping that in mind, the volatility of most of the detected compounds was marginal. Generally, the presented results indicated that all the analyzed BSG samples should be considered low-emission materials, which is beneficial considering their waste origin. 

## 3. Materials and Methods

### 3.1. Materials

The malts applied in the presented study are summarized in Table 6, along with their main properties and additional information. Various properties resulted from the special malting procedures applied, e.g., for the caramel, chocolate, black, or smoked malts. Caramel malts are prepared by controlled drying of wet germinated barley, yielding starch conversion and further sugar caramelization. In some cases, like Vienna or Special B malts, lower kilning temperatures are applied with a simultaneous elongation of the drying time, increasing the extent of the Maillard reactions on account of caramelization. Chocolate malts are relatively similar to black malts, but are roasted at slightly lower temperatures for a shorter time, so the effect is smoother. Smoked malts are prepared similarly to other smoked food products, e.g., meat or fish. Wet germinated barley is dried by smoke from different types of wood. In the case of typical smoked malt, it is beechwood, while for peated malt it is turf. Grodziski malt differs not only in terms of the wood used to generate smoke (oak) but also in the grain used, and it is wheat malt.

Most of the malts applied have their equivalents offered by other producers. All malts were acquired from the online store Twój Browar S.C. (Wrocław, Poland). The malts were already milled by the supplier.

### 3.2. Laboratory-Scale Brewing and BSG Generation

To precisely evaluate the impact of malt recipe on the composition and properties of the generated BSG, the applied BSG samples were generated during laboratory-scale brewing. Mashing, which results in the generation of BSG, was performed according to the modified Institute of Brewing’s 149 °F (65 °C) isothermal mash method [97]. Malts (1 kg) were put into a mashing tun containing water (grain-to-water ratio of 1:3), heated to 70 °C, then kept at 65 °C for 60 min. The mash was heated up to 76 °C for the mashout. Sparging was performed with water heated up to 76 °C (grain-to-water ratio 1:8). After lautering and sparging, BSG was removed and dried at 70 °C for 24 h. Then, it was ground using a Mockmill 100 stone grain mill from Wolfgang Mock GmbH (Groß-Umstadt, Germany).

The following malts have been applied individually: Pilsen, Wheat, Rye, Munich Light, Munich, and Vienna. Moreover, combinations of wheat and rye malts with Pilsen malt were applied at a 1:1 mass ratio. Abbey, Brown, Coffee Light, and Special B malts were introduced into Pilsen malt in 20 wt%, while for Coffee 500, Chocolate 400, Chocolate 900, and Roasted Barley it was in 10 wt% loadings. To simulate the formulations for smoked beers, Smoked, Grodziski, and Peated malts were applied in the 25 and 50 wt% loadings. Table S1 presents the malt recipes used to prepare particular BSG samples.

### 3.3. Characterization Techniques

The color of the prepared BSG samples was assessed according to the Commission Internationale de l’Eclairage (CIE) through L*a*b* coordinates [98] using an EnviSense NR145 colorimeter with the measurement geometry 45°/0° for the assessment of the color standard, as described elsewhere [99]. A detailed description of the applied methodology is provided in Section S2.1 of the Supplementary Data.

Fourier transform infrared spectroscopy (FTIR) was performed using the Jasco (Hachioji, Japan) FT/IR-4600 apparatus in attenuated total reflectance (ATR) mode. FTIR analyses were carried out using 64 scans at a resolution of 4 cm^−1^ in the wavenumber range of 4000–400 cm^−1^. The results were applied for calculations of the total crystallinity index (TCI), lateral order index (LOI), and hydrogen bonding index (HBI), according to the data provided in Section S2.2 of the Supplementary Data.

The crystallinity structure of the BSG samples was determined using an X-ray URD 6 diffractometer (Rich Seifert & CoGmbH, Ahrensburg, Germany) by monochromatic X-ray diffraction with a wavelength of λ = 1.5406 Å (CuKα) in a 2θ angle range from 7.0 to 40.0° with a step of 0.05. The details on the cellulose crystallinity index (CCI) calculations are provided in Section S2.3 of the Supplementary Data.

The chemical composition of BSG, including the contents of carbon, hydrogen, sulfur, nitrogen, and chlorine; the content of ash, the substances soluble in cold water, hot water, and neutral detergents (CWEs, HWEs, and NDEs, respectively); neutral detergent fiber (NDF) content; acid detergent fiber (ADF) content; acid detergent lignin (ADL) content; and the hemicellulose, cellulose, and lignin components, were analyzed in the Department of Genetics, Plant Breeding, and Bioresource Engineering at the University of Warmia and Mazury in Olsztyn following the methodology described by Stolarski et al. [100]. A detailed description of the applied methodology is provided in Section S2.4 of the Supplementary Data.

The antioxidant properties of the BSG samples were measured using a DPPH free radical scavenging assay, as described in our previous work [75]. A detailed description of the applied methodology is provided in Section S2.5 of the Supplementary Data.

A disk diffusion assay has been performed to evaluate the antimicrobial activity of the BSG samples and their toxicity against the selected bacterial strains. It was performed using the method described by Marchel et al. [82] and the following bacterial strains: *E. coli* ATCC 25922, *S. aureus* ATCC 25923, *P. aeruginosa* ATCC 27853, *Staphylococcus epidermidis* ATCC 12228, and *Streptomyces pneumoniae* KBMiM. A detailed description of the applied methodology is provided in Section S2.6 of the Supplementary Data.

The emission studies of volatile organic compounds (VOCs) emitted in the gaseous phase from the investigated BSGs were performed using the Micro-Chamber/Thermal Extractor™ (µ-CTE™ 250, Markes International, Inc., Bridgend, UK) stationary system, equipped with 4 similar stainless-steel chambers (inner volume of 114 cm^3^ each). Detailed information about the characteristics of the µ-CTE™ 250 system might be found elsewhere [101,102]. Before analysis, the BSG samples were placed on Petri dishes and weighed (the average weight of the analyzed samples was 1.067 ± 0.066 g). The chamber conditioning parameters of the investigated samples were as follows: conditioning temperature—50 ± 0.5 °C; conditioning time—30 ± 0.5 min; and nitrogen flow rate—12 ± 0.5 mL/min. The emitted VOCs were purged outside the chamber and adsorbed on a Tenax TA sorption bed installed in a stainless-steel tube (60/80 mesh, Merck KGaA, Darmstadt, Germany). Next, the VOCs collected on Tenax TA were liberated from the sorption medium using a two-stage thermal desorption (TD) system (Markes Series 2 Thermal Desorption Systems; UNITY/TD-100). The separation, identification, and final determination of the emitted VOCs were performed using a gas chromatography technique with a flame ionization detector (GC-FID) (Agilent 7820A GC, Agilent Technologies, Inc., Santa Clara, CA, USA). Detailed information about the thermal extraction conditions and GC-FID working parameters is attached in Section S2.7 of the Supplementary Data. The number of representatives of monoaromatic hydrocarbons (benzene, toluene, ethylbenzene, styrene, and *p,m*-xylene) and terpenes (β-Pinene; Camphene; α-Pinene; 3-Carene; α-Terpinene; (R)-(+)-Limonene; γ-Terpinene L-(−)-Fenchone; Fenchol; (1R)-(+)-Camphor; Isoborneol; Menthol; Citronellol; (+)-Pulegone; Geranyl acetate; α-Cedrene; α-Humulene; Nerolidol; (+)-Cedrol; (−)-α-Bisabolol) was assessed using the external standard calibration method (ESTD). For this purpose, the following reference standard solutions were used: (i) EPA VOC Mix 2, containing 13 VOCs at a concentration of 2000 µg/mL of each, Supelco, USA; and (ii) Cannabis Terpene Mix A, TraceCERT^®^, containing 20 terpenes at a content level of 2000 μg/mL each, Merck KGaA, Darmstadt, Germany. Detailed information about the conditions of the calibration process and the applied devices are described in Section S2.8 of the Supplementary Data.

## 4. Conclusions

The presented study reported an extensive characterization of the various types of BSG generated from a very broad range of malt compositions, mirroring the actual by-products generated in industrial beer production. An unprecedented number of twenty-two different variants of BSG have been analyzed. The analyzed samples mirrored the by-products resulting from the manufacturing of the whole palette of beers from light lagers, through wheat and rye beers, as well as pale, amber, and brown ales, to stouts or porters, including even the application of smoked and peated malts, which also have their followers. In contrast to most of the reported studies, the malt compositions for all of the analyzed samples have been provided. Notably, all the samples were processed in similar conditions, enabling their actual comparison and the drawing of proper conclusions.

The presented results point to noticeable differences in BSG appearance, which fully align with the variations in produced beer. Such a finding may noticeably enhance the management of BSG by facilitating the prediction of its appearance. The chemical composition, antioxidant activity, and structure of BSG have been mostly affected by the type of applied grain and malt kilning procedures. Significant differences, especially for the content of extractives, cellulose, and proteins, related to the grain structure may be potentially essential for further BSG applications in animal or human feeding, but also for its biorefinery. Notably, all the analyzed BSG samples should be considered low-emission materials with very limited antimicrobial activity. Therefore, their harmfulness to human health and the environment is limited, broadening their potential application range.

## Figures and Tables

**Table 1:** The values of color parameters and digital color reproduction of the obtained beer and brewers’ spent grain (BSG) samples.

BSG Sample	Beer								BSG						
	Color, EBC	L*	a*	b*	BI	Chroma	Hue	Color	L*	a*	b*	BI	Chroma	Hue	Color
Pilsen	5.5	84.09	7.29	61.42	13.67	61.85	83.23		66.63	8.48	21.42	12.21	23.04	68.41	
Wheat 50%	6.1	84.09	7.29	61.42	13.67	61.85	83.23		70.37	8.41	18.53	11.08	20.35	65.58	
Wheat 100%	6.8	81.25	11.78	68.38	18.98	69.39	80.23		69.98	8.34	17.96	10.99	19.80	65.08	
Rye 50%	7.2	81.25	11.78	68.38	18.98	69.39	80.23		65.27	8.41	19.44	12.09	21.18	66.60	
Rye 100%	8.8	77.33	16.42	74.76	25.03	76.54	77.61		64.11	8.27	17.35	11.81	19.22	64.52	
Munich I	14.8	67.38	26.67	72.8	38.50	77.53	69.88		65.15	9.63	22.28	13.85	24.27	66.62	
Munich II	20.8	58.50	33.99	65.96	50.59	74.20	62.74		64.29	9.70	22.06	14.07	24.10	66.26	
Vienna	7.5	81.25	11.78	68.38	18.98	69.39	80.23		67.63	8.49	20.95	11.98	22.61	67.93	
Abbey	12.6	74.34	20.47	78.16	30.25	80.80	75.32		63.45	9.14	20.66	13.43	22.60	66.13	
Brown	29.2	47.56	39.57	57.34	66.07	69.67	55.39		54.36	9.95	20.09	16.55	22.42	63.66	
Coffee light	35.2	40.50	40.65	51.57	76.19	65.66	51.75		59.91	10.22	21.87	15.65	24.14	64.96	
Special B	38.7	36.47	42.00	48.38	84.46	64.07	49.04		57.90	10.02	20.75	15.76	23.04	64.21	
Coffee 500	33.9	43.49	41.14	54.11	72.76	67.97	52.75		55.19	9.82	20.58	16.24	22.80	64.49	
Chocolate 400	30.6	45.63	39.69	55.74	68.35	68.43	54.55		55.72	9.81	20.43	16.05	22.66	64.35	
Chocolate 900	51.9	25.62	39.33	37.41	104.34	54.28	43.57		53.21	9.79	19.53	16.58	21.85	63.39	
Roasted barley	62.9	18.76	36.77	27.40	122.23	45.86	36.69		50.08	9.97	19.04	17.75	21.49	62.36	
Smoked 25%	6.6	84.09	7.29	61.42	13.67	61.85	83.23		67.25	8.89	20.93	12.45	22.74	67.00	
Smoked 50%	7.7	81.25	11.78	68.38	18.98	69.39	80.23		65.85	9.28	21.69	13.24	23.59	66.84	
Grodziski 25%	7.1	81.25	11.78	68.38	18.98	69.39	80.23		68.25	8.51	19.37	11.65	21.15	66.28	
Grodziski 50%	8.6	81.25	11.78	68.38	18.98	69.39	80.23		67.73	9.09	19.00	12.28	21.06	64.42	
Peated 25%	5.5	84.09	7.29	61.42	13.67	61.85	83.23		69.38	8.49	21.03	11.69	22.68	68.03	
Peated 50%	5.5	84.09	7.29	61.42	13.67	61.85	83.23		67.28	8.69	21.29	12.30	22.99	67.78	

**Table 2:** Composition of analyzed BSG samples. Asterisk (*) indicates that protein content has been calculated from nitrogen content using a specific protein factor equal to 5.83. The following abbreviations have been applied: cold water extractives (CWEs), hot water extractives (HWEs), neutral detergent extractives (NDEs), neutral detergent fiber (NDF), acid detergent fiber (ADF), and Trolox equivalent antioxidant capacity (TEAC). Values in parentheses indicate standard deviation.

BSG Sample	Content, %_DW_										Elemental Content, %_DW_					Trolox_eq_, mg/L	TEAC, mg/g_DW_
	CWEs	HWEs	NDEs	NDF	ADF	Hemicellulose	Cellulose	Lignin	Ash	Protein *	C	H	N	S	Cl		
Pilsen	41.04 (1.32)	48.01 (1.19)	10.49 (0.65)	41.50 (0.53)	12.90 (0.01)	28.60 (0.53)	9.67 (0.10)	3.22 (0.10)	3.05 (0.01)	19.75 (0.02)	51.29 (0.41)	7.55 (0.12)	3.16 (0.01)	0.143 (0.001)	0.073 (0.003)	22.36 (8.36)	0.89 (0.33)
Wheat 50%	51.80 (0.01)	58.99 (0.19)	10.96 (0.50)	30.05 (0.30)	9.69 (0.44)	20.36 (0.13)	7.27 (0.31)	2.42 (0.13)	2.65 (0.08)	23.06 (0.29)	50.42 (0.67)	7.75 (0.06)	3.69 (0.05)	0.157 (0.001)	0.027 (0.009)	18.11 (7.36)	0.72 (0.29)
Wheat 100%	52.41 (0.28)	60.69 (0.45)	9.64 (0.08)	29.67 (0.37)	7.52 (0.18)	22.15 (0.19)	5.33 (0.16)	2.20 (0.02)	2.11 (0.03)	22.99 (0.20)	49.24 (0.46)	7.37 (0.18)	3.68 (0.03)	0.169 (0.002)	0.025 (0.014)	10.48 (2.37)	0.42 (0.09)
Rye 50%	52.03 (0.85)	58.13 (1.83)	9.56 (0.55)	32.32 (1.28)	9.81 (0.67)	22.50 (0.61)	6.68 (0.57)	3.13 (0.10)	2.75 (0.07)	21.11 (0.07)	50.88 (0.66)	7.61 (0.12)	3.38 (0.01)	0.141 (0.003)	0.030 (0.004)	21.76 (2.69)	0.87 (0.11)
Rye 100%	58.85 (0.28)	63.74 (1.07)	7.86 (0.29)	28.40 (0.78)	8.78 (0.67)	19.62 (0.11)	5.59 (0.54)	3.19 (0.13)	2.59 (0.03)	19.66 (0.02)	50.26 (0.63)	7.48 (0.11)	3.14 (0.01)	0.139 (0.009)	0.047 (0.004)	21.42 (5.89)	0.86 (0.24)
Munich I	54.91 (0.83)	61.34 (0.51)	6.03 (0.07)	32.63 (0.44)	8.60 (0.18)	24.03 (0.62)	6.59 (0.04)	2.01 (0.10)	2.37 (0.15)	17.93 (0.16)	46.72 (0.46)	6.47 (0.13)	2.87 (0.03)	0.176 (0.004)	0.014 (0.002)	16.86 (3.71)	0.67 (0.15)
Munich II	52.41 (0.04)	59.82 (0.02)	6.51 (0.42)	33.68 (0.40)	10.38 (0.16)	23.30 (0.24)	7.61 (0.09)	2.77 (0.06)	2.56 (0.02)	19.79 (0.32)	47.11 (0.35)	6.51 (0.12)	3.17 (0.05)	0.200 (0.004)	0.020 (0.001)	20.66 (11.19)	0.83 (0.45)
Vienna	52.00 (0.09)	58.18 (0.06)	7.65 (0.16)	34.16 (0.22)	9.69 (0.15)	24.48 (0.06)	7.29 (0.01)	2.40 (0.16)	2.63 (0.05)	16.97 (0.01)	46.56 (0.31)	6.43 (0.09)	2.72 (0.01)	0.170 (0.004)	0.023 (0.001)	18.69 (3.62)	0.75 (0.15)
Abbey	47.91 (0.22)	53.89 (0.36)	9.35 (0.01)	36.76 (0.37)	10.31 (0.42)	26.45 (0.05)	7.45 (0.19)	2.80 (0.23)	2.63 (0.05)	18.06 (0.15)	48.00 (0.10)	6.67 (0.05)	2.89 (0.02)	0.180 (0.002)	0.015 (0.004)	13.14 (1.34)	0.53 (0.05)
Brown	52.14 (0.37)	56.95 (0.42)	7.58 (0.05)	35.47 (0.47)	13.13 (0.20)	22.34 (0.27)	7.77 (0.23)	5.36 (0.02)	2.63 (0.01)	18.47 (0.21)	48.13 (0.20)	6.67 (0.20)	2.96 (0.03)	0.180 (0.007)	0.026 (0.003)	15.70 (2.96)	0.63 (0.12)
Coffee light	55.46 (0.13)	61.95 (0.08)	7.56 (0.35)	30.49 (0.28)	9.98 (0.41)	20.51 (0.13)	7.17 (0.23)	2.81 (0.17)	2.43 (0.07)	17.65 (0.25)	48.15 (0.47)	6.68 (0.14)	2.82 (0.04)	0.172 (0.002)	0.034 (0.004)	19.79 (2.67)	0.79 (0.11)
Special B	50.64 (0.50)	56.49 (0.28)	7.09 (0.16)	36.42 (0.13)	11.40 (0.46)	25.02 (0.33)	6.94 (0.18)	4.46 (0.28)	2.53 (0.03)	18.67 (0.11)	47.97 (0.03)	6.70 (0.01)	2.99 (0.02)	0.172 (0.003)	0.060 (0.005)	19.10 (11.87)	0.76 (0.47)
Coffee 500	52.29 (0.08)	57.63 (0.20)	7.06 (0.32)	35.31 (0.12)	12.66 (0.10)	22.65 (0.02)	7.57 (0.19)	5.09 (0.08)	2.66 (0.09)	17.35 (0.06)	47.56 (0.62)	6.57 (0.13)	2.78 (0.01)	0.171 (0.001)	0.021 (0.003)	18.22 (4.55)	0.73 (0.18)
Chocolate 400	52.88 (0.18)	57.57 (0.79)	7.08 (0.58)	35.35 (0.21)	12.64 (0.13)	22.71 (0.34)	8.32 (0.11)	4.32 (0.24)	2.73 (0.04)	17.49 (0.27)	48.04 (0.37)	6.66 (0.11)	2.80 (0.04)	0.175 (0.001)	0.028 (0.002)	18.94 (9.86)	0.76 (0.39)
Chocolate 900	48.64 (0.16)	53.12 (0.38)	8.19 (0.07)	38.69 (0.31)	12.98 (0.18)	25.71 (0.13)	7.76 (0.03)	5.22 (0.21)	2.77 (0.01)	18.40 (0.03)	49.01 (0.01)	6.72 (0.05)	2.94 (0.01)	0.179 (0.002)	0.037 (0.002)	17.56 (6.26)	0.70 (0.25)
Roasted barley	48.57 (0.34)	54.28 (0.01)	8.51 (0.35)	37.20 (0.34)	12.72 (0.33)	24.48 (0.02)	8.18 (0.25)	4.54 (0.07)	2.89 (0.03)	17.69 (0.09)	48.08 (0.09)	6.57 (0.01)	2.83 (0.01)	0.172 (0.002)	0.016 (0.004)	15.42 (6.83)	0.62 (0.27)
Smoked 25%	43.26 (0.40)	50.13 (0.33)	10.56 (0.21)	39.31 (0.12)	10.46 (0.10)	28.85 (0.22)	7.26 (0.24)	3.20 (0.14)	2.63 (0.01)	19.10 (0.17)	48.23 (0.43)	6.68 (0.02)	3.06 (0.03)	0.190 (0.001)	0.037 (0.002)	19.06 (1.55)	0.76 (0.06)
Smoked 50%	43.04 (0.29)	49.61 (0.28)	11.72 (0.15)	38.67 (0.13)	10.11 (0.09)	28.56 (0.04)	6.30 (0.17)	3.81 (0.08)	2.77 (0.08)	20.12 (0.06)	48.84 (0.18)	6.81 (0.01)	3.22 (0.01)	0.195 (0.001)	0.010 (0.004)	17.63 (1.34)	0.71 (0.05)
Grodziski 25%	47.45 (0.41)	56.99 (0.19)	13.30 (0.31)	29.71 (0.11)	7.82 (0.43)	21.89 (0.32)	5.40 (0.29)	2.43 (0.14)	2.16 (0.03)	17.79 (0.09)	47.60 (0.08)	6.72 (0.05)	2.85 (0.01)	0.179 (0.001)	0.018 (0.001)	16.30 (6.99)	0.65 (0.28)
Grodziski 50%	49.62 (0.17)	59.65 (0.60)	11.01 (0.16)	29.34 (0.43)	7.77 (0.05)	21.57 (0.48)	5.38 (0.06)	2.39 (0.01)	2.12 (0.02)	18.16 (0.11)	48.28 (0.04)	6.77 (0.21)	2.91 (0.02)	0.184 (0.003)	0.030 (0.002)	11.68 (2.68)	0.47 (0.11)
Peated 25%	46.16 (0.19)	52.57 (0.18)	9.19 (0.06)	38.24 (0.12)	10.17 (0.11)	28.07 (0.23)	7.54 (0.13)	2.63 (0.02)	2.51 (0.05)	17.92 (0.01)	47.94 (0.10)	6.71 (0.06)	2.87 (0.01)	0.179 (0.004)	0.032 (0.002)	16.50 (0.14)	0.66 (0.01)
Peated 50%	42.84 (0.23)	48.64 (0.09)	7.89 (0.21)	43.48 (0.12)	11.48 (0.20)	32.00 (0.08)	8.18 (0.09)	3.30 (0.11)	2.70 (0.05)	18.78 (0.19)	49.18 (0.39)	6.82 (0.02)	3.01 (0.03)	0.198 (0.003)	0.039 (0.002)	13.06 (2.02)	0.52 (0.08)

**Table 3:** Comparison between the chemical composition of BSG samples reported in the presented study and the literature data.

Content, %_DW_						Reference
NDF	Hemicellulose	Cellulose	Lignin	Ash	Proteins	
-	-	-	-	3.8	26.9	[39]
-	-	-	-	3.4	21.6–26.4	[37]
-	-	-	-	3.3	26.7	[34]
-	25.4	21.8	11.9	2.4	24.0	[35]
-	16.8	28.4	27.8	4.6	-	[27]
51.0	-	-	-	4.1	23.4	[30]
43.5	-	-	-	4.1	22.6	[28]
42.2	-	-	-	4.7	22.8	[29]
48.2	-	22.2	-	-	22.1	[36]
-	21.7	19.3	19.4	4.2	24.7	[31]
-	12.0	40.2	11.5	3.3	14.2	[32]
-	22.2	26.8	14.1	-	-	[26]
-	21.9	29.6	21.7	1.2	24.6	[33]
24.0–40.1	-	-	-	3.1–3.4	15.2–23.9	[13]
43.0	-	-	-	3.6	31.0	[38]
-	-	-	-	3.3–4.3	22.2–30.2	[11]
41.5	28.6	9.7	3.2	3.1	19.8	Presented study (Pilsen malt)
28.4–43.5	19.6–32.0	5.3–9.7	2.0–5.2	2.1–3.1	17.0–23.1	Presented study (all samples)

**Table 4:** The results of the performed disk diffusion assay for the analyzed BSG samples, including visual presentation, the existence of a bacterial growth inhibition zone (BGIZ), and a zone of more intense bacterial growth (ZMIBG). Numbers (1–5) refer to the applied strains: 1—*E. coli* strain ATCC 25922, 2—*P. aeruginosa* strain ATCC 27853, 3—*S. aureus* strain ATCC 25923, 4—*S. epidermidis* strain ATCC 12228, and 5—*S. pneumoniae* strain KBMiM. P points to the presence of pigment in ZMIBG. Minus sign (−) means not observed, while plus sign (+) means observed.

BSG Sample	Presentation	BGIZ, mm					ZMIBG				
	*E. coli, P. aeruginosa, S. aureus, S. epidermidis, S. pneumoniae*	1	2	3	4	5	1	2	3	4	5
Control agar disk without BSG	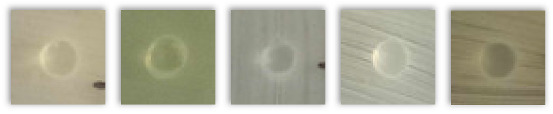	−	−	−	−	−	−	−	−	−	−
Pilsen	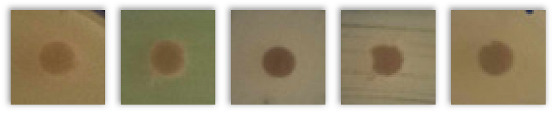	−	−	−	−	−	+	− P	+	+	+ P
Wheat 50%	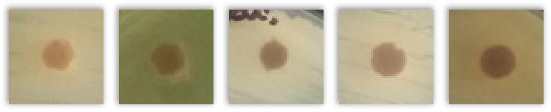	−	−	−	−	−	+	− P	+	+	+ P
Wheat 100%	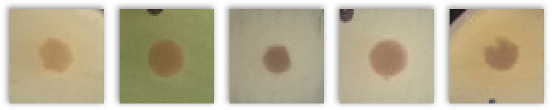	−	−	−	−	−	+	− P	+	+	+ P
Rye 50%	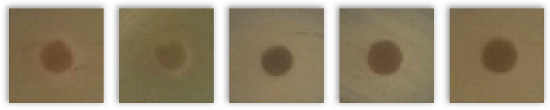	−	−	−	−	−	+	−	+	+	+ P
Rye 100%	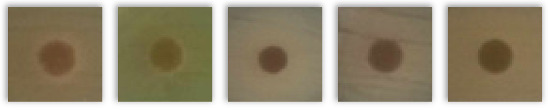	−	−	−	−	−	+	− P	+	+	+ P
Munich I	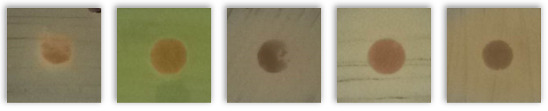	−	−	−	−	−	+	− P	+	+	+ P
Munich II	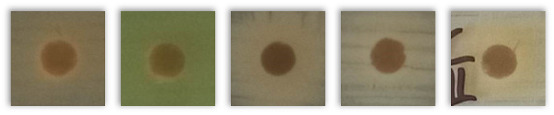	−	−	−	−	−	+	− P	+	+	+ P
Vienna	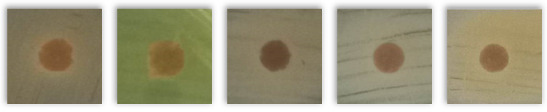	−	−	−	−	−	+	− P	+	+	+ P
Abbey	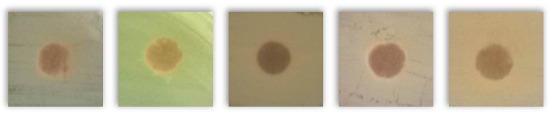	−	−	−	−	−	+	− P	+	+	+ P
Brown	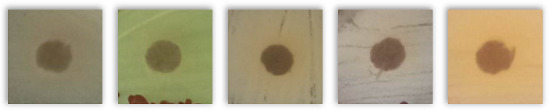	−	−	−	−	−	+	− P	+	+	+ P
Coffee light	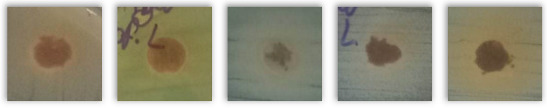	−	−	−	−	−	+	− P	+	+	+ P
Special B	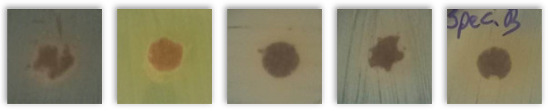	−	−	−	−	−	+	− P	+	+	+ P
Coffee 500	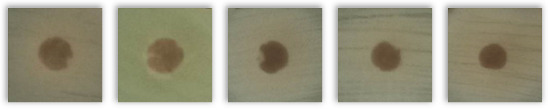	−	−	−	−	−	+	−	+	+	+ P
Chocolate 400	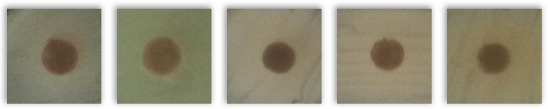	−	−	−	−	−	+	−	+	+	+ P
Chocolate 900	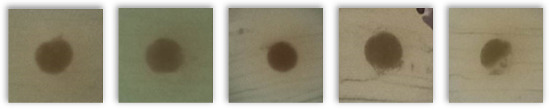	−	−	−	−	−	+	− P	+	+	+ P
Roasted barley	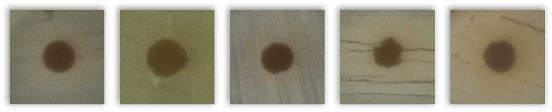	−	−	−	−	−	+	−	+	+	+ P
Smoked 25%	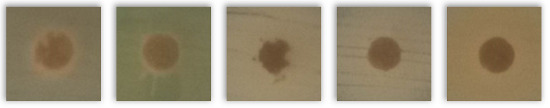	−	−	−	−	−	+	− P	+	+	+ P
Smoked 50%	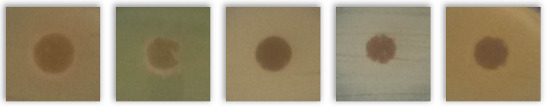	−	−	−	−	−	+	− P	+	+	+ P
Grodziski 25%	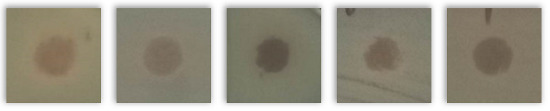	−	−	−	−	−	+	−	+	+	+ P
Grodziski 50%	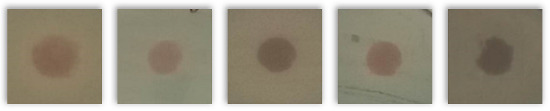	−	−	−	−	−	+	−	+	+	+ P
Peated 25%	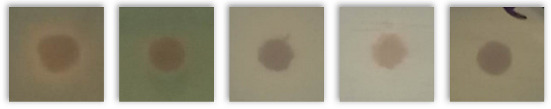	−	−	−	−	−	+	+ P	+	+	+ P
Peated 50%	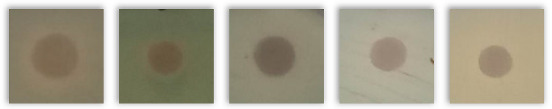	−	−	−	−	−	+	+ P	+	+	+ P

**Table 5:** Quantitative data on volatile organic compound (VOC) emissions from the analyzed BSG samples. L_D_ indicates values below the limit of detection (0.5 ng/g), while L_Q_ indicates values below the limit of quantification (1.5 ng/g).

BSG Sample	TVOC	Benzene	Toluene	Ethylbenzene	*p,m*-Xylene	Styrene	α-Pinene	Camphene	β-Pinene	3-Carene + α-Terpinene	(*R*)-(+)-Limonene	γ-Terpinene	L-(-)-Fenchone	Fenchol	(1R)-(+)-Camphor	Isoborneol + DL-Menthol	(+/−)-β-Citronellol	(R)-(+)-Pulegone	Geranyl Acetate	α-Cedrene	α-Humulene	Nerolidol 1	(+)-Cedrol	α-Bisabolol
	ng/g																							
Pilsen	1647	15.0	L_D_	L_D_	L_D_	L_D_	L_Q_	5.6	L_D_	L_D_	3.8	6.5	L_D_	L_D_	8.3	16.0	L_D_	L_D_	L_Q_	L_D_	L_D_	L_D_	L_D_	L_D_
Wheat 50%	1626	16.6	L_Q_	L_D_	L_D_	L_D_	27.7	35.2	L_Q_	L_D_	6.4	L_D_	L_D_	L_D_	4.7	20.2	L_D_	L_D_	L_Q_	L_Q_	L_D_	L_D_	L_D_	L_D_
Wheat 100%	1434	11.0	L_D_	L_D_	L_D_	L_D_	5.0	L_D_	L_D_	1.8	7.4	2.3	L_D_	L_D_	L_Q_	9.6	L_D_	L_Q_	L_D_	L_D_	L_D_	L_D_	L_D_	L_D_
Rye 50%	1997	12.4	L_D_	L_D_	L_Q_	L_D_	L_Q_	12.6	L_Q_	L_D_	6.3	L_Q_	L_D_	4.1	L_D_	13.2	L_D_	L_Q_	L_Q_	L_D_	L_D_	L_D_	L_D_	L_D_
Rye 100%	1844	11.4	L_D_	L_D_	L_D_	L_D_	L_Q_	L_D_	L_D_	L_D_	6.9	2.3	L_D_	L_D_	L_D_	11.4	L_D_	14.2	L_D_	L_D_	L_D_	L_D_	L_D_	L_D_
Munich I	2617	11.5	L_D_	L_D_	L_D_	L_D_	2.6	16.0	4.5	L_D_	7.7	2.4	L_D_	L_D_	L_Q_	17.3	L_D_	4.4	L_D_	L_D_	L_D_	L_D_	L_D_	L_D_
Munich II	5889	16.0	L_Q_	L_D_	3.6	10.3	34.9	15.8	L_Q_	2.1	8.4	3.3	L_D_	L_D_	L_D_	4.8	L_D_	L_Q_	8.4	L_D_	L_D_	L_D_	L_D_	L_D_
Vienna	1240	12.2	L_Q_	L_D_	L_Q_	4.2	4.2	18.1	L_D_	L_D_	5.8	L_Q_	L_D_	L_D_	11.7	23.8	L_Q_	L_Q_	L_Q_	L_Q_	L_D_	L_D_	L_D_	L_D_
Abbey	2289	12.4	L_D_	L_D_	L_D_	L_D_	10.6	21.2	L_D_	2.3	14.1	2.4	L_D_	4.4	7.7	10.5	L_D_	8.5	5.0	8.8	L_D_	L_D_	L_D_	L_D_
Brown	2195	26.7	L_Q_	L_D_	2.9	6.0	L_D_	L_D_	L_D_	L_D_	L_Q_	L_D_	L_D_	L_D_	5.2	28.5	L_D_	L_D_	L_Q_	1.9	L_D_	L_D_	L_D_	L_D_
Coffee light	2526	23.4	L_Q_	L_D_	3.9	L_D_	17.8	9.3	L_Q_	L_D_	7.4	L_D_	L_D_	L_D_	L_D_	15.2	L_D_	L_Q_	L_Q_	L_Q_	L_D_	L_D_	L_D_	L_D_
Special B	1276	13.9	L_D_	L_D_	L_D_	5.3	L_Q_	5.1	L_D_	1.1	8.5	2.4	L_D_	7.0	3.2	11.0	L_D_	15.3	L_D_	L_Q_	L_D_	L_D_	L_D_	L_D_
Coffee 500	1263	10.8	L_Q_	L_D_	2.4	3.1	L_D_	26.7	L_D_	L_D_	8.8	L_D_	L_D_	L_D_	5.9	12.0	L_D_	4.0	9.7	L_Q_	L_D_	L_D_	L_D_	L_D_
Chocolate 400	1469	19.1	L_Q_	L_D_	L_D_	L_D_	L_D_	8.1	L_Q_	L_D_	12.2	L_D_	L_D_	L_D_	L_D_	17.0	L_D_	L_Q_	4.4	L_D_	L_D_	L_D_	L_D_	L_D_
Chocolate 900	1783	15.3	L_D_	L_D_	L_D_	L_D_	2.6	L_D_	L_D_	L_D_	11.4	28.1	L_D_	4.3	3.5	13.3	L_D_	L_D_	L_Q_	L_D_	L_D_	L_D_	L_D_	L_D_
Roasted barley	1428	13.0	14.5	L_D_	L_D_	8.4	8.8	12.3	L_D_	L_D_	4.5	7.3	L_D_	L_D_	14.6	18.1	L_D_	4.6	L_D_	L_D_	L_D_	L_D_	L_D_	L_D_
Smoked 25%	1779	14.4	16.7	L_D_	L_D_	5.7	5.8	24.1	L_D_	L_D_	7.2	25.9	5.8	5.2	2.0	24.8	10.1	3.8	5.0	28.9	11.6	L_D_	9.1	L_D_
Smoked 50%	1849	15.0	17.1	L_D_	L_D_	5.8	6.1	25.5	L_D_	L_D_	7.4	26.5	6.0	5.4	2.1	25.7	10.3	3.9	5.1	29.5	12.0	L_D_	9.3	L_D_
Grodziski 25%	1284	12.6	L_D_	L_D_	L_D_	L_D_	4.7	15.8	L_D_	L_D_	7.9	12.8	L_D_	6.6	7.0	14.6	L_D_	3.9	L_D_	L_D_	L_D_	L_D_	L_D_	L_D_
Grodziski 50%	892	9.9	L_D_	L_D_	L_D_	L_D_	L_D_	5.7	L_D_	L_D_	4.0	27.8	L_D_	L_D_	L_D_	11.8	L_D_	L_Q_	L_D_	L_D_	L_D_	L_D_	L_D_	L_D_
Peated 25%	1637	12.4	4.3	L_D_	L_D_	L_D_	6.7	24.3	L_D_	L_D_	6.9	12.9	L_D_	L_D_	6.3	24.8	L_D_	4.6	6.5	L_D_	5.4	L_D_	L_D_	L_D_
Peated 50%	1492	13.8	L_D_	L_D_	L_D_	5.6	7.2	L_D_	L_Q_	L_D_	5.4	14.6	3.7	7.2	4.1	16.7	L_Q_	5.3	L_D_	L_Q_	L_D_	L_D_	10.6	L_D_

**Table 6:** The list of applied malts, along with their characteristics.

Name	Producer	Extract, %	Color, EBC	Total Protein, %	Max. Kilning Temperature, °C	Additional Information
Pilsen	La Malterie du Château SA (Beloeil, Belgium)	>82.0	3.5	<12.0	80–85	-
Wheat	Palatia Malz GmbH (Kreimbach-Kaulbach, Germany)	>82.0	3.6–6.0	<13.5	80–85 *	-
Rye	Viking Malt Sp. z o.o. (Sierpc, Poland)	>80	4.0–10.0	<11 *	72–80	-
Munich Light	La Malterie du Château SA (Beloeil, Belgium)	>80	13–17	<12	100–105	-
Munich	La Malterie du Château SA (Beloeil, Belgium)	>80	21–28	<12	100–105	-
Vienna	La Malterie du Château SA (Beloeil, Belgium)	>80	4.0–7.0	<12	85–90	-
Abbey	La Malterie du Château SA (Beloeil, Belgium)	>78	41–49	<11.5	110	-
Brown	Thomas Fawcett & Sons Ltd. (Castleford, UK)	>70.0	175–200	<11.6	175 *	-
Cafe Light	La Malterie du Château SA (Beloeil, Belgium)	>77	220–280	<11.5 *	200	-
Special B	La Malterie du Château SA (Beloeil, Belgium)	>77	260–320	<12.5 *	93 *	-
Cafe	La Malterie du Château SA (Beloeil, Belgium)	>75.5	420–520	<11.5 *	220	-
Czekoladowy jasny	Viking Malt Sp. z o.o. (Sierpc, Poland)	>68	350–450	<11.5 *	220 *	-
Czekoladowy ciemny	Viking Malt Sp. z o.o. (Sierpc, Poland)	>67	800–1000	<11.5 *	220 *	-
Roasted barley	La Malterie du Château SA (Beloeil, Belgium)	>65	1000–1400	<11.6 *	230	-
Smoked	La Malterie du Château SA (Beloeil, Belgium)	>77	4.0–12.0	<11.5	85 *	Phenols 1.6–4.0 ppm
Grodziski	Viking Malt Sp. z o.o. (Sierpc, Poland)	>81	8.0–12.0	<13.5	85 *	Phenols 5–10 ppm
Peated	La Malterie du Château SA (Beloeil, Belgium)	>81	3.5	<11.7	85 *	Phenols 5–10 ppm

* indicates data for another manufacturer’s equivalent or based on the literature reports.

## Data Availability

Data will be made available on request.

## Supplementary Materials

The following supporting information can be downloaded at https://www.mdpi.com/article/10.3390/molecules30132809/s1: Figure S1: FTIR spectra for the particular brewers’ spent grain samples; Figure S2: XRD diffractograms for the particular brewers’ spent grain samples; Table S1: Malt recipes applied during laboratory-scale brewing yielding particular brewers’ spent grain samples; Table S2: TD-GC-FID system working parameters used to investigate the emissions of VOCs from the studied BSG samples; Table S3: Values of cellulose crystallinity index (CCI) calculated from XRD results along with total crystallinity index (TCI), lateral order index (LOI), and hydrogen bonding index (HBI) determined by FTIR analysis; Table S4: Pearson correlation coefficients characterizing the impact of BSG chemical composition and color parameters on the cellulose crystallinity index, total crystallinity index, lateral order index, and hydrogen bonding index; Table S5: Pearson correlation coefficients characterizing the impact of BSG (only the samples composed of barley malts) chemical composition and color parameters on the cellulose crystallinity index, total crystallinity index, lateral order index, and hydrogen bonding index; and Table S6: List of VOCs emitted from BSG samples along with the information on occupational safety and health hazards. References [103,104,105,106,107,108,109,110,111] are cited in the supplementary materials.
