# Supplementary material for: Brewers’ Spent Grain from Different Types of Malt: A Comprehensive Evaluation of Appearance, Structure, Chemical Composition, Antimicrobial Activity, and Volatile Emissions
Source: Molecules. 2025 Jun 30;30(13):2809. doi: 10.3390/molecules30132809 (PMC12251032; doi:10.3390/molecules30132809)
Supplement: Supplementary file 1 [file molecules-30-02809-s001.zip › molecules-3702803-supplementary.pdf]

molecules

Supplementary Materials

Brewers' Spent Grain from Different Types of Malt: A Comprehensive Evaluation of Appearance, Structure, Chemical Composition, Antimicrobial Activity, and Volatile Emissions

Aleksander Hejna [1,2,*], Joanna Aniśko-Michalak [1], Katarzyna Skórczewska [3], Mateusz Barczewski [1], Paweł Sulima [4], Jerzy Andrzej Przyborowski [4], Hubert Cieśliński [5], and Mariusz Marć [6]

[1] Institute of Materials Technology, Poznan University of Technology, Piotrowo 3, 61-138 Poznań, Poland; aleksander.hejna@put.poznan.pl (A.H.), joanna.anisko@put.poznan.pl (J.A.), mateusz.barczewski@put.poznan.pl (M.B.)
[2] Department of Polymer Technology, Gdańsk University of Technology, Narutowicza 11/12, 80-233 Gdańsk, Poland; aleksander.hejna@put.poznan.pl (A.H.)
[3] Faculty of Chemical Technology and Engineering, Bydgoszcz University of Science and Technology, Seminaryjna 3 Street, 85-326 Bydgoszcz, Poland; Katarzyna.Skorczewska@pbs.edu.pl (K.S.)
[4] Department of Genetics, Plant Breeding and Bioresource Engineering, University of Warmia and Mazury in Olsztyn, Plac Łódzki 3, 10-724 Olsztyn, Poland; pawel.sulima@uwm.edu.pl (P.S.), jerzy.przyborowski@uwm.edu.pl (J.A.P)
[5] Department of Molecular Biotechnology and Microbiology, Gdańsk University of Technology, Narutowicza 11/12, 80-233 Gdańsk, Poland; hcieslin@pg.edu.pl (H.C.)
[6] Department of Analytical Chemistry, Gdańsk University of Technology, Narutowicza 11/12, 80-233 Gdańsk, Poland; mariusz.marc@pg.edu.pl (M..M.)

* Correspondence: aleksander.hejna@put.poznan.pl

S1. Laboratory-scale brewing and BSG generation

Table S1. Malt recipes applied during laboratory-scale brewing, yielding particular brewers' spent grain samples.

BSG sample	Primary malt	Content, wt%	Secondary malt	Content, wt%
Pilsen	Pilsen	100	-	-
Wheat 50%	Wheat	50	Pilsen	50
Wheat 100%	Wheat	100	-	-
Rye 50%	Rye	50	Pilsen	50
Rye 100%	Rye	100	-	-
Munich I	Munich Light	100	-	-
Munich II	Munich	100	-	-
Vienna	Vienna	100	-	-
Abbey	Pilsen	80	Abbey	20
Brown	Pilsen	80	Brown	20
Coffee light	Pilsen	80	Cafe Light	20
Special B	Pilsen	80	Special B	20
Coffee 500	Pilsen	90	Cafe	10
Chocolate 400	Pilsen	90	Czekoladowy jasny	10
Chocolate 900	Pilsen	90	Czekoladowy ciemny	10
Roasted barley	Pilsen	90	Roasted barley	10
Smoked 25%	Pilsen	75	Smoked	25
Smoked 50%	Pilsen	50	Smoked	50
Grodziski 25%	Pilsen	75	Grodziski	25
Grodziski 50%	Pilsen	50	Grodziski	50
Peated 25%	Pilsen	75	Peated	25
Peated 50%	Pilsen	50	Peated	50

S2. Characterization techniques

S2.1. Brewers' spent grain color evaluation

In the CIELab system, L* stands for the color lightness (L*=0 for black and L*=100 for white), a* for the green(-) / red(+) axis, and b* for the blue(-) / yellow(+) axis). Based on the obtained values, the following color parameters have been determined:

- The total color difference parameter (ΔE*) [1] was calculated according to formula (S1):

$$\Delta E^* = [(\Delta L^*)^2 + (\Delta a^*)^2 + (\Delta b^*)^2]^{0.5} \qquad (S1)$$

- Chroma (C*) was calculated according to formula (S2):

$$C^* = [(a^*)^2 + (b^*)^2]^{0.5} \qquad (S2)$$

- Hue was calculated according to formula (S3):

$$hue = \tan^{-1}\frac{b^*}{a^*} \qquad (S3)$$

- The browning index (BI) was calculated according to formulas (S4 and S5) [2]:

$$BI = \frac{100\cdot(x-0.31)}{0.17} \qquad (S4)$$

$$x = \frac{a^*+1.75\cdot L^*}{5.645\cdot L^*+a^*-0.3012\cdot b^*} \qquad (S5).$$

Moreover, to present the digital reproduction of the color of the brewers' spent grain, the determined parameters were converted to the commonly used Adobe RGB color space defined by the three chromaticities of the red, green, and blue additive primaries [3]. The

determined CIELab parameters were converted to the normalized CIEXYZ space according to the following formulas (S6-S10) [4]:

$$X = X_w f^{-1}\left(\frac{L^*+16}{116} + \frac{a^*}{500}\right) \quad \text{(S6)}$$

$$Y = Y_w f^{-1}\left(\frac{L^*+16}{116}\right) \quad \text{(S7)}$$

$$Z = Z_w f^{-1}\left(\frac{L^*+16}{116} - \frac{b^*}{200}\right) \quad \text{(S8)}$$

where:

$$f^{-1}(t) = \begin{cases} t^3 & t > \delta \\ 3\delta^2\left(t - \frac{4}{29}\right) & otherwise \end{cases} \quad \text{(S9)}$$

and

$$\delta = \frac{6}{29} \quad \text{(S10)}$$

and X_w, Y_w, and Z_w are the values for the reference white point, which for Iluminant D65 equal 0.950450, 1.000000, and 1.088754, respectively, according to ITU-R Recommendation BT.709.

Then, the obtained values were further converted to linear RGB (u), according to the following formulas (S11-S22):

$$\begin{bmatrix} r \\ g \\ b \end{bmatrix} = [M]^{-1} \begin{bmatrix} X \\ Y \\ Z \end{bmatrix} \quad \text{(S11)}$$

where

$$[M] = \begin{bmatrix} S_r X_r & S_g X_g & S_b X_b \\ S_r Y_r & S_g Y_g & S_b Y_b \\ S_r Z_r & S_g Z_g & S_b Z_b \end{bmatrix} \quad \text{(S12)}$$

where

$$X_r = \frac{x_r}{y_r} \quad \text{(S13)}$$

$$Y_r = 1 \quad \text{(S14)}$$

$$Z_r = \frac{(1-x_r-y_r)}{y_r} \quad \text{(S15)}$$

$$X_g = \frac{x_g}{y_g} \quad \text{(S16)}$$

$$Y_g = 1 \quad \text{(S17)}$$

$$Z_g = \frac{(1-x_g-y_g)}{y_g} \quad \text{(S18)}$$

$$X_b = \frac{x_b}{y_b} \quad \text{(S19)}$$

$$Y_b = 1 \quad \text{(S20)}$$

$$Z_b = \frac{(1-x_b-y_b)}{y_b} \quad \text{(S21)}$$

$$\begin{bmatrix} S_r \\ S_g \\ S_b \end{bmatrix} = \begin{bmatrix} X_r & X_g & X_b \\ Y_r & Y_g & Y_b \\ Z_r & Z_g & Z_b \end{bmatrix}^{-1} \begin{bmatrix} X_w \\ Y_w \\ Z_w \end{bmatrix} \quad \text{(S22)}$$

and (x_r, y_r), (x_g, y_g), and (x_b, y_b) are the chromaticity coordinates of the RGB system, which in the case of Adobe RGB are (0.64, 0.33), (0.21, 0.71), and (0.15, 0.06), while X_w, Y_w, and Z_w are the values for the reference white point, as mentioned above [5].

Further, the conversion from linear RGB (u) to nonlinear RGB (V) was performed using the gamma companding technique, according to the following formula (S23):

$$V = \upsilon^{\frac{1}{\gamma}} \quad \text{(S23)}$$

where g stands for the gamma value characteristic to a color system; in the case of Adobe RGB, it equals 2.2.

The obtained RGB values were in the nominal range [0.0, 1.0]. To present them in the most commonly used range of [0, 255], the components were multiplied by 255.

S2.2. Fourier transform infrared spectroscopy (FTIR) of brewers' spent grain

The results of the FTIR analysis were applied for the calculations of the total crystallinity index (TCI), lateral order index (LOI), and hydrogen bonding index (HBI), according to the following equations (S24-S26):

$$TCI = \frac{a_{1371}}{a_{2900}} \quad (S24)$$

$$LOI = \frac{a_{1430}}{a_{895}} \quad (S25)$$

$$HBI = \frac{a_{3336}}{a_{1336}} \quad (S26)$$

where a_x is the absorbance at a particular wavelength, while x stands for the wavelength in cm^{-1}.

S2.3. X-ray diffraction analysis (XRD) of brewers' spent grain

Based on the XRD results, the cellulose crystallinity index (CCI) was calculated using the following equation (S27) (Segal et al., 1959):

$$CCI = 100 \cdot \frac{I_{002} - I_{am}}{I_{002}} \quad (S27)$$

where I_{002} is the maximum intensity of the (002) plane diffraction at 2θ = 22-23° and I_{am} is the intensity of the peak at 2θ = 18°.

S2.4. Chemical composition of brewers' spent grain

The ash content in the BSG samples was determined in an Eltra TGA-Thermostep automatic analyzer (Germany) using the PN-EN ISO 18122:2016-01 standard.

The elemental composition of BSG, and the contents of carbon (C), hydrogen (H), and sulfur (S) were determined using an Eltra CHS-500 automatic analyzer (Germany) following the PN-EN ISO 16948:2015-07 and PN-EN ISO 16994:2016-10 standards. In addition, the total nitrogen (N) was determined by the Kjeldahl method using the K-435 mineralization apparatus and the Buchi B-324 distillery apparatus (Switzerland). The chlorine content was determined according to the PN-ISO 587:2000 standard. After the samples burned in a Nabertherm muffle furnace (Germany) at a temperature of 650 °C in the presence of an Eschka's mixture, the sample of an aqueous extract was titrated with 0.025 mol/L $AgNO_3$.

The content of the soluble substances in cold water (CWEs) was determined by weight based on the differences in the mass of the samples before and after the extraction. The samples placed in F57 filtration bags (ANKOM Technology) were extracted with distilled water (20–25 °C) for 48 h. Then, they were rinsed twice in the ANKOM A200 apparatus (USA) and dried before weighing (105 °C). The weighed samples were used for further analyses. Similarly, the content of the soluble substances in hot water (HWEs) was determined. In this case, the samples were extracted for 3 h at 100 °C and dried prior to weighing at 105 °C. An analytical balance with accuracy to 0.1 mg was used to weigh the samples.

After the determination of substances soluble in hot water, laboratory analyses were performed to determine the neutral detergent fiber (NDF) (PN-EN ISO 16472:2007), acid detergent fiber (ADF), and acid detergent lignin (ADL) fractions (PN-EN ISO 13906:2009) in the biomass using the Ankom A200 extraction system. On the basis of the differences in the obtained results, the contents of the neutral detergent extractives (NDEs), hemicellulose, cellulose, and lignin in the tested samples was calculated. The following equations (S28-S36) have been applied during the analyses:

$$CWE = 100 - \left(100 \cdot \frac{W_{CW} - W_1}{W_2}\right) \quad (S28)$$

$$HWE = 100 - \left(100 \cdot \frac{W_{HW} - W_1}{W_2}\right) \quad (S29)$$

$$NDF = \frac{100 \cdot (W_{NDF} - W_1 \cdot C)}{W_2} \quad (S30)$$

$$ADF = \frac{100 \cdot (W_{ADF} - W_1 \cdot C)}{W_2} \quad (S31)$$

$$ADL = \frac{100 \cdot (W_{ADL} - W_1 - W_A)}{W_2} \quad \text{(S32)}$$
$$NDE = 100 - HWE - NDF \quad \text{(S33)}$$
$$Hemicellulose = NDF - ADF \quad \text{(S34)}$$
$$Cellulose = ADF - ADL \quad \text{(S35)}$$
$$Lignin = ADL \quad \text{(S36)}$$

where W_1 is the bag tare weight, g; W_2 is the sample weight, g; W_{CW} is the dried weight of the bag and samples after extraction in cold water, g; W_{HW} is the dried weight of the bag and samples after extraction in hot water, g; W_{NDF} is the dried weight of the bag with fiber after NDF extraction, g; W_{ADF} is the dried weight of the bag with fiber after ADF extraction, g; C is the blank bag correction (the running average of the final oven-dried weight divided by the original blank bag weight); W_{ADL} is the dried weight of the bag with fiber after ADL extraction, g; and W_A is the weight of residue after the incineration of the filter bag and samples at 520 °C, g.

S2.5. Antioxidant activity of brewers' spent grain

The antioxidant properties of the BSG samples were measured using a 2,2-diphenyl-1-picrylhydrazyl (DPPH) free radical scavenging assay. DPPH is a stable organic nitrogen radical. The scavenging of free radicals by antioxidants causes the fading of a DPPH solution in methanol, which is monitored by a UV–Vis spectrophotometer. To investigate the antioxidant activity of the applied by-products, they were extracted with methanol at a concentration of 2 g/l (50 ml of methanol per 100 mg of material). The extraction process was performed using a magnetic stirrer at an ambient temperature with a rotation speed of 300 rpm for 30 min. The vacuum filtration station was equipped with quantitative medium filter papers to filter the extract. The prepared extracts were further diluted with methanol to obtain extracts in concentrations 2, 1, 0.5, 0.1, and 0.05 g/l. These extracts were immediately added to the 63 μM DPPH solution in methanol [6]. To proceed with the DPPH assay, 0.15 ml of the extracts and 2.85 ml of the DPPH solution were added to the dark flask. They were kept closed in the dark for 30 min before measurement, which was performed using a spectrophotometer UV–Vis UviLine 9400 from SI Analytics GmbH (Germany) at 517 nm [7]. The antioxidant activity of each extract is presented as an inhibition I calculated using the following equation (S37):

$$I = \frac{A_{control} - A_{sample}}{A_{control}} \quad \text{(S37)}$$

where $A_{control}$ is the absorbance of the DPPH solution at 517 nm; and A_{sample} is the absorbance of the sample at 517 nm.

The calculation of I enabled the determination of IC50, the concentration of the extract for which it shows 50% maximum inhibition. Finally, the DPPH scavenging activity values for the samples were presented as Trolox equivalent antioxidant capacity (TEAC) in mg of Trolox per dry mass of the sample.

S2.6. Antimicrobial activity of brewers' spent grain

A disk diffusion assay was performed to evaluate the antimicrobial activity of the BSG samples. First, BSG-filled agar disks were prepared. The 0.75 g of bacteriological agar and 6.25 g of the tested BSG were placed into a 100 ml conical flask. Then, 25 ml of redistilled water was added to the flask and stirred until uniform. The obtained mixture of agar and BSG in water was autoclaved under a pressure of 1.5 atm and 121 °C. After autoclaving, the sterile mixture was poured into a Petri dish (9 cm in diameter) under the same conditions as described above. After the agar and BSG mixtures hardened, agar disks with a diameter of 6 mm were cut out using a sterile dermatological die. The disk diffusion assay for the analyzed BSG toxicity studies against the selected bacterial strains was performed using the method described by Marchel et al. [8] with the following

modifications. In this study, in addition to the strains of bacteria *E. coli* ATCC 25922, *S. aureus* ATCC 25923, and *P. aeruginosa* ATCC 27853, the bacterial strains of *Staphylococcus epidermidis* ATCC 12228 and *Streptomyces pneumoniae* KBMiM were also used. Due to contamination risk during disk preparation, they were sterilized using a UV lamp before being transferred using sterile tweezers to Petri dishes with the inoculated bacteria on the LB-agar medium (A&A Biotechnology, Poland). For this purpose, the agar disks' lower and upper bases were illuminated with UV light (λ = 234 nm) for 5 minutes. The sterilization process using a UV lamp was carried out in a laminar chamber MSC Advantage 1.2 (Thermo Fischer Scientific, US). Thereafter, the inoculated plates with attached discs were incubated for 24 h at 37 °C in the incubator StabiliTherm (Thermo Fischer Scientific, US). After the incubation, the presence or absence of zones of bacterial growth inhibition around the disks was checked. If there was a bacterial growth inhibition zone around the disk, its diameter was measured to assess the antimicrobial properties of each tested spent grain.

S2.7. Assessment of volatile organic compound (VOC) emissions from brewers' spent grain

Detailed information about the working parameters of the applied analytical equipment, TD-GC-FID, is listed in Table S2.

Table S2. TD-GC-FID system working parameters used to investigate the emissions of VOCs from the studied BSG samples.

**Working conditions of the two-stage thermal desorption unit**	
Analytical procedure acronym	TD-GC-FID
Applied thermal desorber	Markes' Series 2 Thermal Desorption System; UNITY/TD-100
Tool for collecting analyte samples from the gaseous phase	A steel tube filled with the sorption medium (Tenax TA), with 60/80 mesh
Steel tube heating time and temperature at the 1st stage of thermal desorption	280 °C for 12 min
Inert gas (He) flow rate through the steel tube	50 mL/min
Microtrap (multi-bed glass tube) temperature during the 1st stage of thermal desorption	0 °C
Microtrap heating time and temperature at the 2nd stage of thermal desorption	300 °C for 5 min
Flow rate of the inert gas (He) through the microtrap to the chromatographic column	2.0 mL/min
**Working conditions of the final determination system**	
Gas chromatograph	Agilent 7820A GC
Detector	Flame ionization detector, detector temp. 850 °C
Transfer line temperature TD-GC	180 °C
Capillary column	DB-1 (J&W), 30 m × 0.32 mm × 5 µm
Helium gas (flow rate)	2.0 mL/min

Temperature program	60 °C for 1 min<br>10 °C/min up to 260 °C, held for 8 min
Data collecting software	OpenLAB CDS ChemStation Workstation VL

S2.8. Calibration of the TD/GC/FID system and QA/QC characteristics

To estimate the TVOC parameter, following the literature data, unidentified peaks were quantified in terms of toluene equivalents (using the FID response factor of toluene) [9,10]. The following solvents and reference solutions (as the external standards) were applied to assess the mass of the analytes emitted from the investigated samples and adsorbed on a Tenax TA sorption medium: (i) methanol for GC (MS SupraSolv®, Merck KGaA, Darmstadt, Germany); (ii) Cannabis Terpene Mix A certified reference material, TraceCERT® (Merck KGaA, Darmstadt, Germany) containing 20 terpenes (β-Pinene; Camphene; α-Pinene; 3-Carene; α-Terpinene; (R)-(+)-Limonene; γ-Terpinene L-(-)-Fenchone; Fenchol; (1R)-(+)-Camphor; Isoborneol; Menthol; Citronellol; (+)-Pulegone; Geranyl acetate; α-Cedrene; α-Humulene; Nerolidol; (+)-Cedrol; (-)-α-Bisabolol) dissolved in MeOH at a content level of 2000 μg/mL each; (iii) EPA VOC Mix 2 (Supelco, USA) containing 13 VOCs (including benzene, toluene, ethylbenzene, styrene, and *p,m*-xylene) dissolved in MeOH at a concentration of 2000 μg/mL each. The calibration process of the TD/GC/FID unit was performed according to the protocol previously published in the literature [11–13].

To perform the calibration, five calibration reference solutions (for a five-point calibration curve) in MeOH were prepared in the concentration range of 2 μg/mL to 120 μg/mL for each analyte. Each time, 1 μL of the prepared calibration solution was injected into the clean Tenax TA sorbent and then analyzed under the same equipment conditions and working parameters as the actual samples. Each point of the calibration curve was repeated three times. The limits of detection (L_D) were evaluated based on the signal-to-noise ratio. The limits of quantification (L_Q) were calculated as 3 × L_D. The average value of L_D was 0.5 ng, and L_Q was 1.5 ng per tube.

S3. Results and discussion

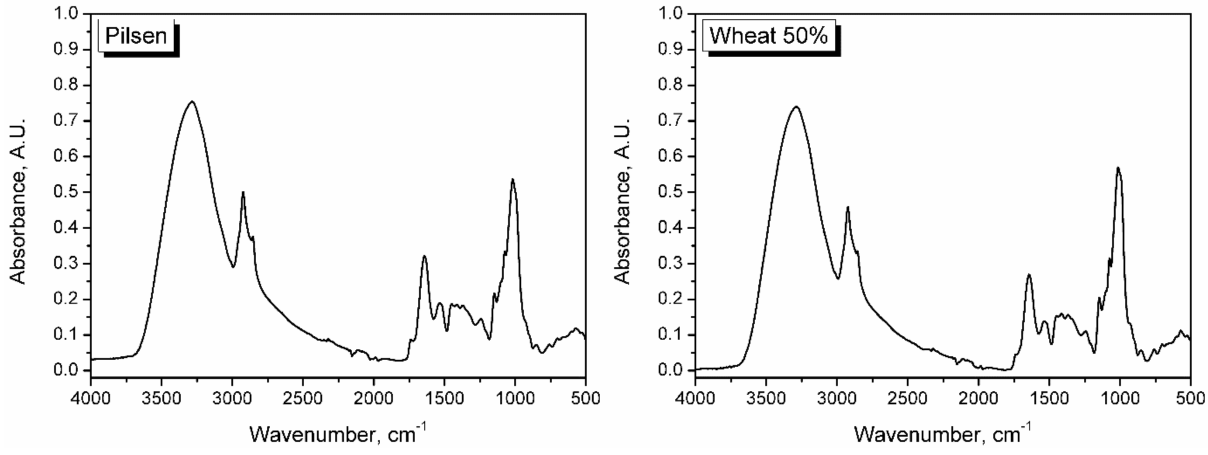

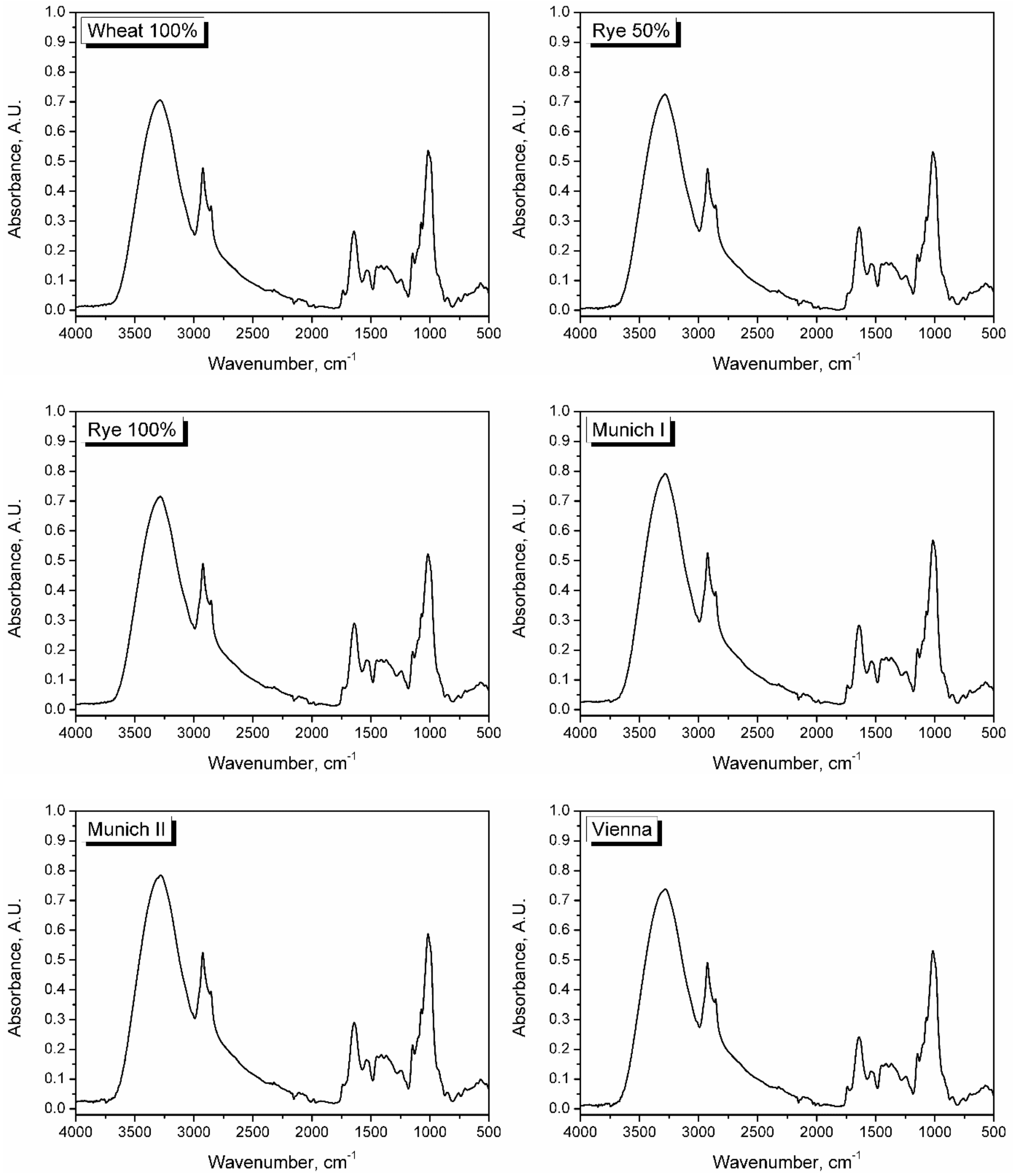
Wheat 100%
Rye 50%
Rye 100%
Munich I
Munich II
Vienna
Absorbance, A.U.
Wavenumber, cm-1
0.0
0.1
0.2
0.3
0.4
0.5
0.6
0.7
0.8
0.9
1.0
4000
3500
3000
2500
2000
1500
1000
500

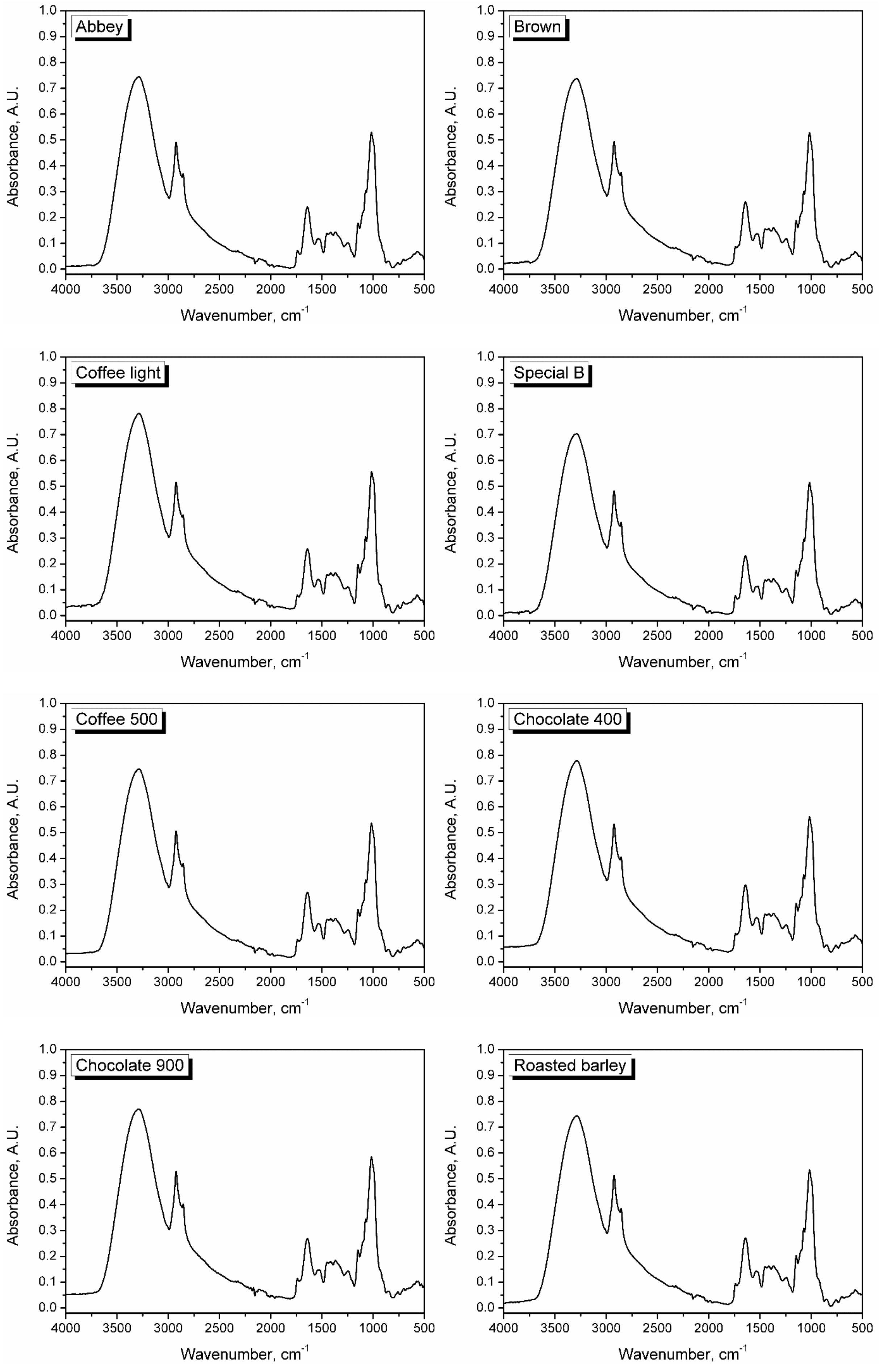
Abbey
Brown
Coffee light
Special B
Coffee 500
Chocolate 400
Chocolate 900
Roasted barley
Absorbance, A.U.
Wavenumber, cm-1

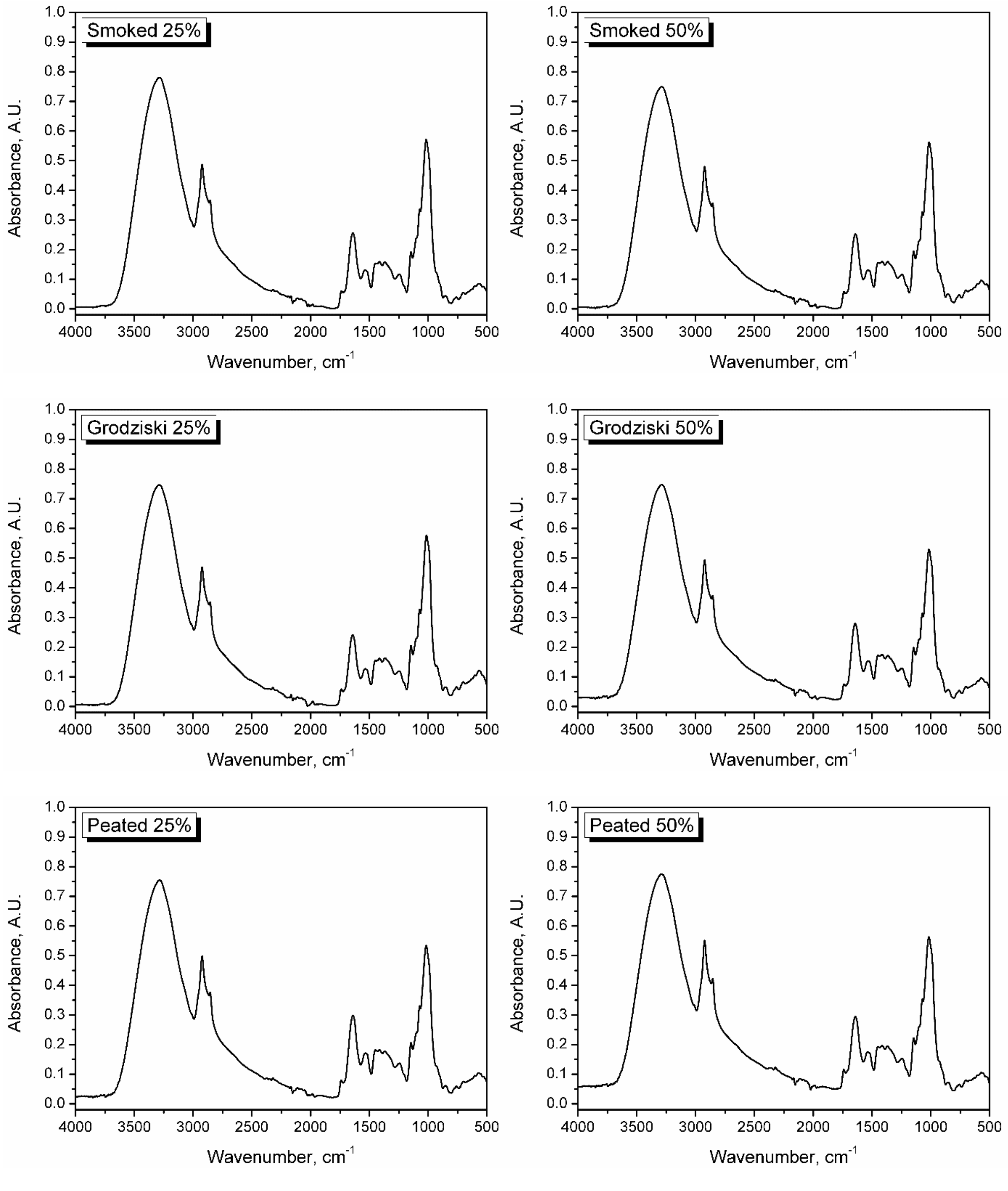

Figure S1. FTIR spectra for the particular brewers' spent grain samples.

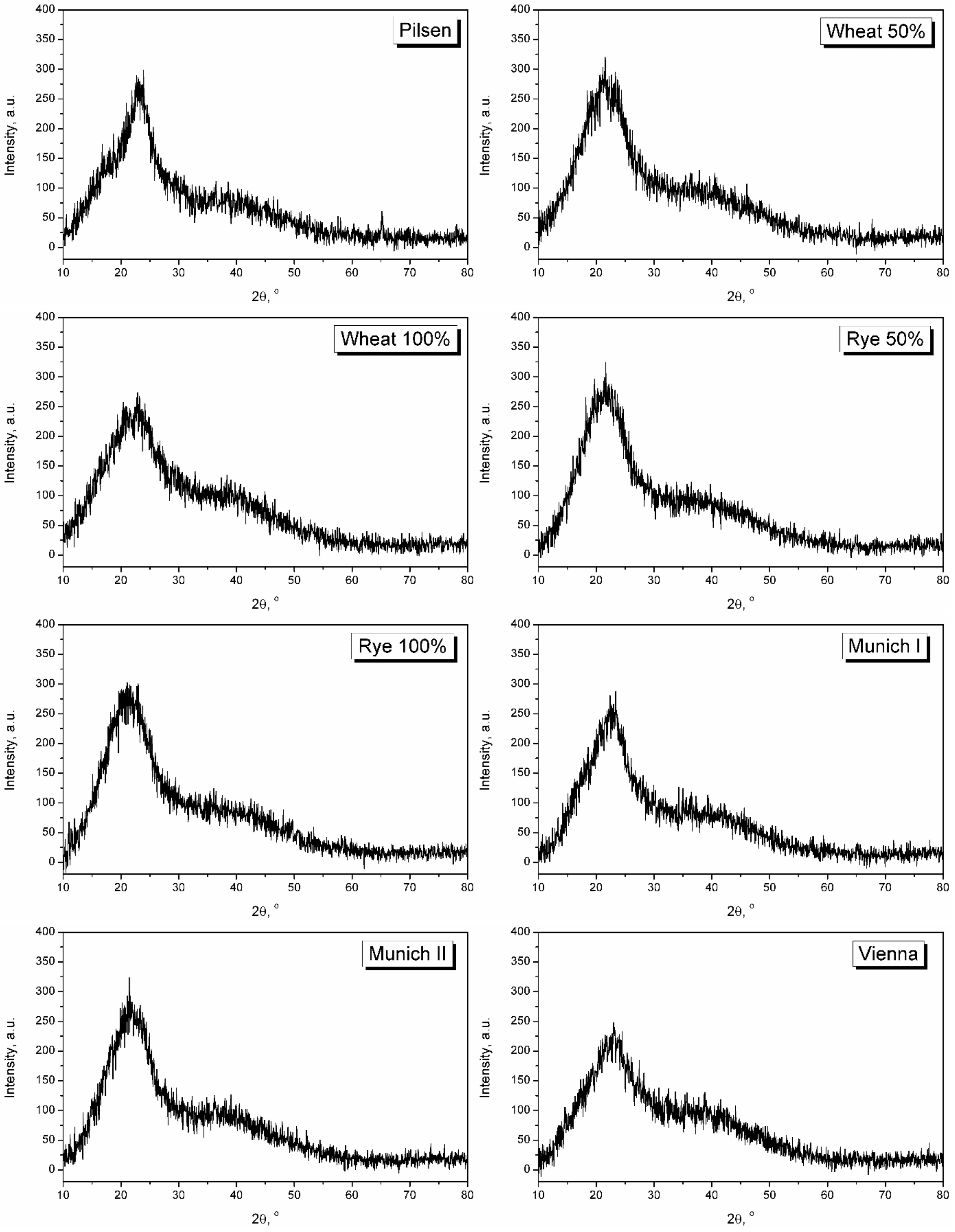
Pilsen
Wheat 50%
Wheat 100%
Rye 50%
Rye 100%
Munich I
Munich II
Vienna
Intensity, a.u.
2θ, °

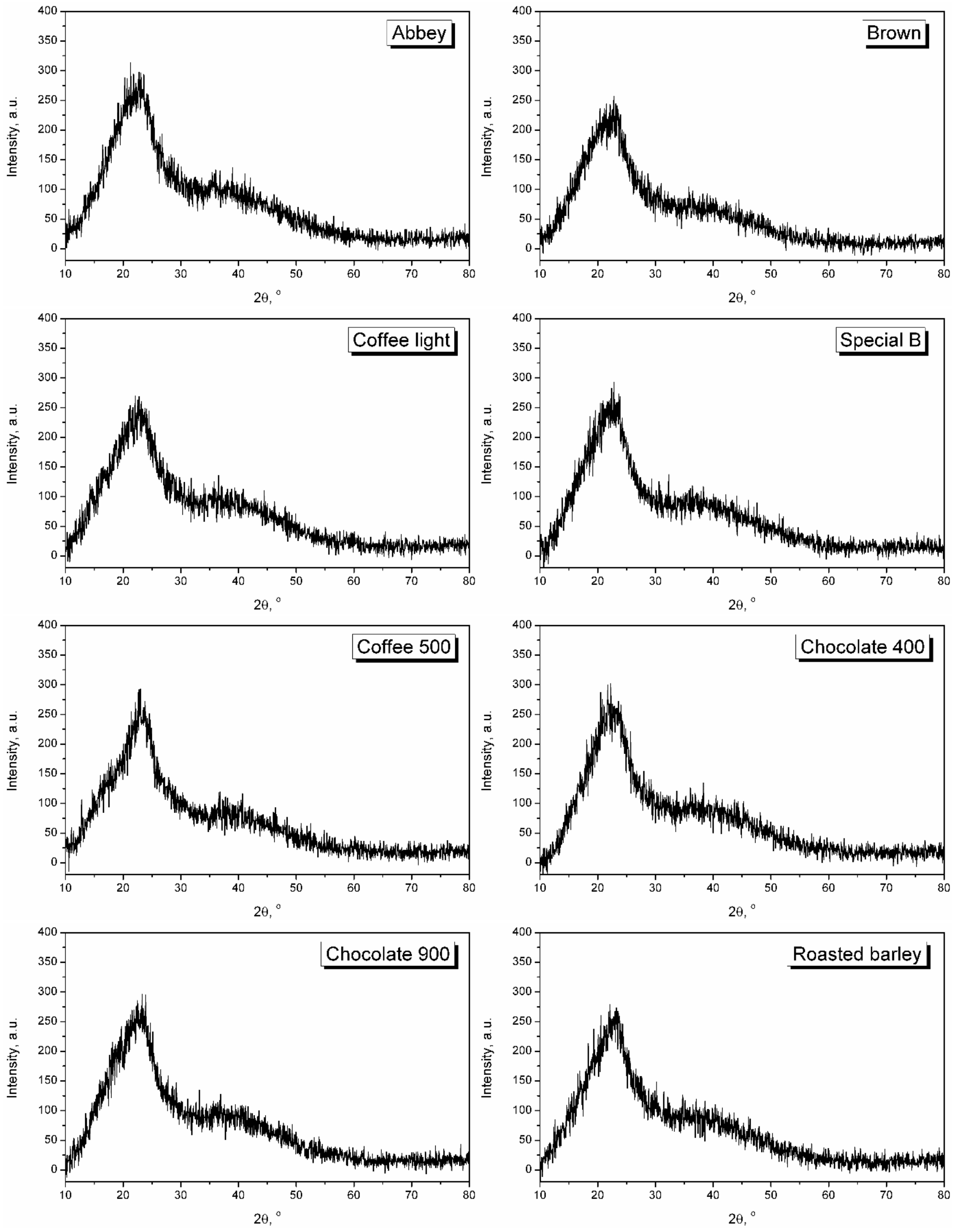
Abbey
Brown
Coffee light
Special B
Coffee 500
Chocolate 400
Chocolate 900
Roasted barley
Intensity, a.u.
2θ, °

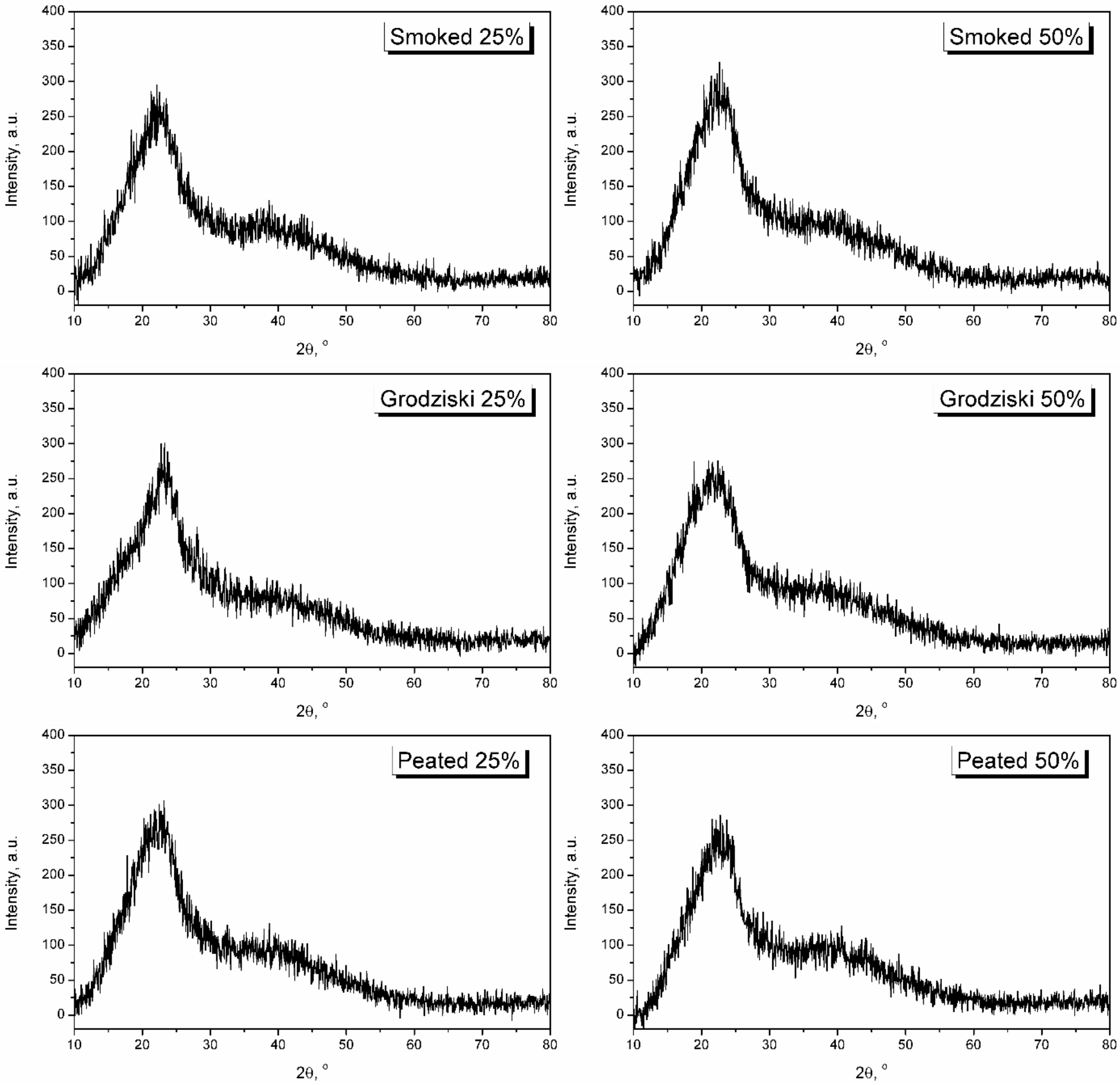

Figure S2. XRD diffractograms for the particular brewers' spent grain samples.

Table S3. Values of cellulose crystallinity index (CCI) calculated from XRD results along with total crystallinity index (TCI), lateral order index (LOI), and hydrogen bonding index (HBI) determined by FTIR analysis.

BSG sample	CCI	TCI	LOI	HBI
Pilsen	0.450	0.443	1.939	4.375
Wheat 50%	0.354	0.411	1.940	4.954
Wheat 100%	0.279	0.382	2.116	5.049
Rye 50%	0.267	0.415	2.109	4.590
Rye 100%	0.277	0.406	2.228	4.871
Munich I	0.329	0.400	2.215	4.792
Munich II	0.352	0.410	2.168	4.681
Vienna	0.337	0.374	2.152	5.224
Abbey	0.359	0.355	2.203	5.717
Brown	0.350	0.390	2.486	5.048
Coffee light	0.370	0.382	2.419	5.054

Special B	0.294	0.364	2.190	5.390
Coffee 500	0.410	0.401	2.109	4.762
Chocolate 400	0.450	0.423	1.982	4.414
Chocolate 900	0.430	0.418	1.809	4.479
Roasted barley	0.459	0.387	2.524	4.933
Smoked 25%	0.386	0.388	1.965	5.276
Smoked 50%	0.309	0.401	2.036	4.932
Grodziski 25%	0.267	0.419	1.694	4.767
Grodziski 50%	0.254	0.415	2.052	4.532
Peated 25%	0.395	0.431	2.061	4.430
Peated 50%	0.423	0.430	1.859	4.167

Table S4. Pearson correlation coefficients characterizing the impact of BSG chemical composition and color parameters on the cellulose crystallinity index, total crystallinity index, lateral order index, and hydrogen bonding index.

	CCI	TCI	LOI	HBI
Cellulose	**0.885**	0.237	0.058	-0.242
Hemicellulose	0.452	0.176	-0.316	-0.170
Lignin	0.454	-0.034	0.189	-0.056
Protein	-0.325	0.105	-0.169	0.010
CWE	-0.350	-0.305	0.480	0.198
HWE	-0.521	-0.272	0.386	0.192
Beer color in EBC	0.457	-0.245	0.421	0.069
Beer L*	-0.424	0.262	-0.449	-0.079
BSG L*	-0.470	0.213	-0.455	-0.053
BSG BI	0.488	-0.250	0.479	0.076

Table S5. Pearson correlation coefficients characterizing the impact of BSG (only the samples composed of barley malts) chemical composition and color parameters on the cellulose crystallinity index, total crystallinity index, lateral order index, and hydrogen bonding index.

	CCI	TCI	LOI	HBI
Cellulose	**0.800**	0.561	-0.206	-0.510
Hemicellulose	0.139	0.367	<u>-0.638</u>	-0.258
Lignin	0.277	0.025	0.053	-0.086
Protein	-0.177	0.296	-0.323	-0.145
CWE	-0.267	-0.385	0.548	0.221
HWE	-0.336	-0.397	0.569	0.252
Beer color in EBC	0.283	-0.206	0.400	0.047
Beer L*	-0.208	0.221	-0.427	-0.054
BSG L*	-0.308	0.189	-0.402	-0.053
BSG BI	0.194	-0.232	0.476	0.082

Table S6. List of VOCs emitted from BSG samples along with the information on occupational safety and health hazards.

Compound	Chemical structure	Formula	NFPA F	NFPA H	NFPA I	GHS pictograms	Vapor pressure at 25 °C, kPa	Flash point, °C
Aromatic hydrocarbons								
Benzene	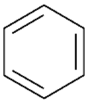	C_6H_6	3	2	0	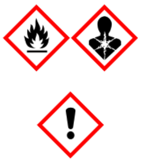	12.60	-11
Toluene	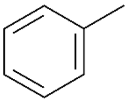	C_7H_8	3	2	0	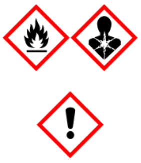	3.79	4
Styrene	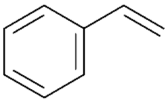	C_8H_8	3	2	2	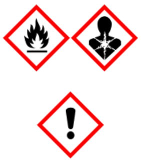	0.85	31
Ethylbenzene	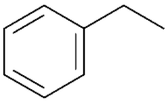	C_8H_{10}	3	2	0	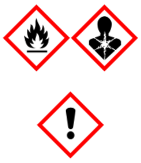	1.28	15
m-Xylene	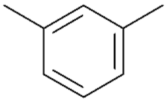	C_8H_{10}	3	2	0	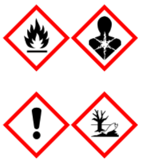	1.33	27
p-Xylene	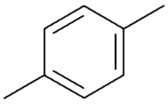	C_8H_{10}	3	2	0	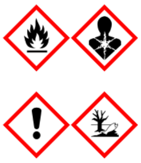	1.18	25
Terpenes and terpenoids								
α-Pinene	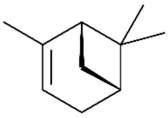	$C_{10}H_{16}$	3	2	0	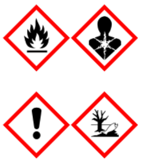	0.63	33
Camphene	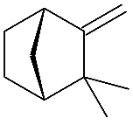	$C_{10}H_{16}$	3	2	0	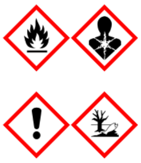	0.33	34
β-Pinene	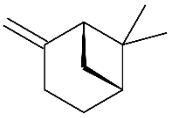	$C_{10}H_{16}$	3	1	0	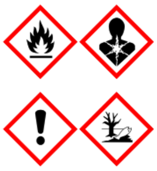	0.39	33

3-Carene	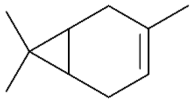	$C_{10}H_{16}$	2	2	0	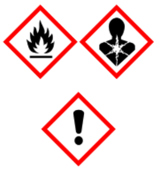	0.50	46
α-Terpinene	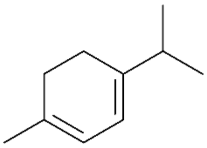	$C_{10}H_{16}$	3	2	0	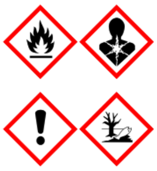	0.22	46
(R)-(+)-Limonene	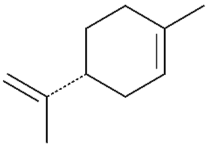	$C_{10}H_{16}$	2	3	0	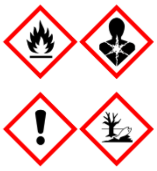	0.21	50
γ-Terpinene	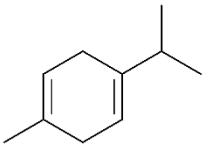	$C_{10}H_{16}$	3	2	2	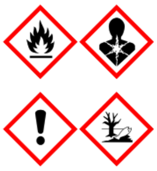	0.10	56
L-(-)-Fenchone	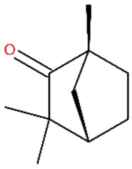	$C_{10}H_{16}O$	2	0	0	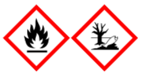	0.22	52
Fenchol	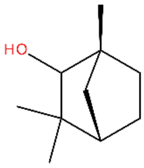	$C_{10}H_{18}O$	2	1	0	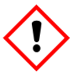	0.009	73
(1R)-(+)-Camphor	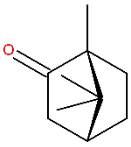	$C_{10}H_{16}O$	2	2	0	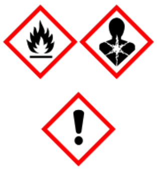	0.05	64
Isoborneol	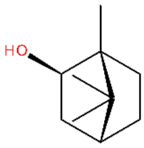	$C_{10}H_{18}O$	2	2	3	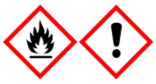	0.005	74
DL-Menthol	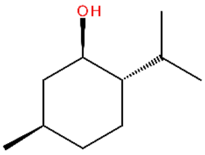	$C_{10}H_{20}O$	2	1	0	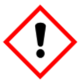	0.015	92
(+/-)-β-Citronellol	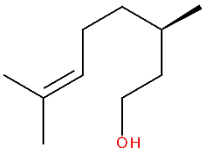	$C_{10}H_{20}O$	2	2	0	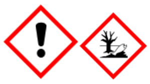	0.003	79

(R)-(+)-Pulegone	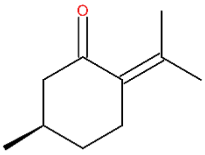	$C_{10}H_{16}O$	2	2	0	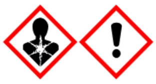	0.016	82
Geranyl Acetate	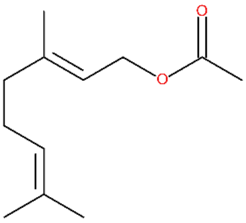	$C_{12}H_{20}O_2$	1	1	0	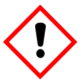	0.003	110
α-Cedrene	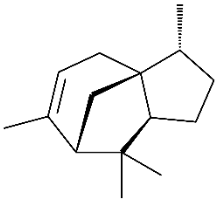	$C_{15}H_{24}$	1	2	1	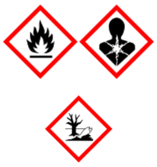	0.002	104
α-Humulene	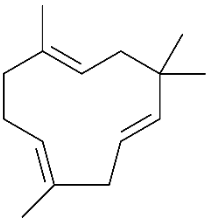	$C_{15}H_{24}$	1	2	0	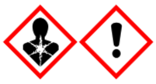	0.002	90
Nerolidol 1	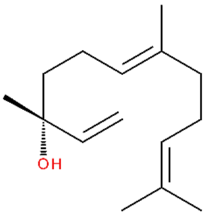	$C_{15}H_{26}O$	1	2	0	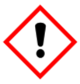	0.002	105
(+)-Cedrol	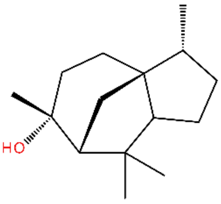	$C_{15}H_{26}O$	0	1	0	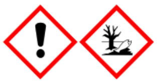	$1·10^{-4}$	116
α-Bisabolol	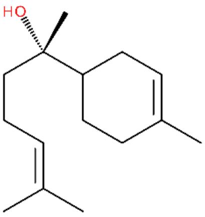	$C_{15}H_{26}O$	1	1	0	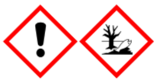	$5·10^{-6}$	148

References

1. Bociaga, E.; Trzaskalska, M. Influence of Polymer Processing Parameters and Coloring Agents on Gloss and Color of Acrylonitrile-Butadiene-Styrene Terpolymer Moldings. *Polimery* 2016, *61*, 544–550, doi:10.14314/polimery.2016.544.
2. Hejna, A.; Marć, M.; Kowalkowska-Zedler, D.; Pladzyk, A.; Barczewski, M. Insights into the Thermo-Mechanical Treatment of Brewers' Spent Grain as a Potential Filler for Polymer Composites. *Polymers (Basel)* 2021, *13*, doi:10.3390/polym13060879.
3. Hunt, R.W.G. *The Reproduction of Colour*; Wiley, 2004; ISBN 9780470024256.
4. López, F.; Valiente, J.M.; Baldrich, R.; Vanrell, M. Fast Surface Grading Using Color Statistics in the CIE Lab Space. In; 2005; pp. 666–673.
5. ADOBE SYSTEMS INCORPORATED Adobe® RGB (1998) Color Image Encoding.
6. Rojas-Lema, S.; Torres-Giner, S.; Quiles-Carrillo, L.; Gomez-Caturla, J.; Garcia-Garcia, D.; Balart, R. On the Use of Phenolic Compounds Present in Citrus Fruits and Grapes as Natural Antioxidants for Thermo-Compressed Bio-Based High-Density Polyethylene Films. *Antioxidants* 2020, *10*, 14, doi:10.3390/antiox10010014.
7. Moreira, M.M.; Morais, S.; Carvalho, D.O.; Barros, A.A.; Delerue-Matos, C.; Guido, Luís.F. Brewer's Spent Grain from Different Types of Malt: Evaluation of the Antioxidant Activity and Identification of the Major Phenolic Compounds. *Food Research International* 2013, *54*, 382–388, doi:10.1016/j.foodres.2013.07.023.
8. Marchel, M.; Cieśliński, H.; Boczkaj, G. Thermal Instability of Choline Chloride-Based Deep Eutectic Solvents and Its Influence on Their Toxicity─Important Limitations of DESs as Sustainable Materials. *Ind Eng Chem Res* 2022, *61*, 11288–11300, doi:10.1021/acs.iecr.2c01898.
9. Massold, E.; Bähr, C.; Salthammer, T.; Brown, S.K. Determination of VOC and TVOC in Air Using Thermal Desorption GC-MS – Practical Implications for Test Chamber Experiments. *Chromatographia* 2005, *62*, 75–85, doi:10.1365/s10337-005-0582-z.
10. Formela, K.; Marć, M.; Namieśnik, J.; Zabiegała, B. The Estimation of Total Volatile Organic Compounds Emissions Generated from Peroxide-Cured Natural Rubber/Polycaprolactone Blends. *Microchemical Journal* 2016, *127*, 30–35, doi:10.1016/j.microc.2016.02.001.
11. Marć, M.; Namieśnik, J.; Zabiegała, B. The Miniaturised Emission Chamber System and Home-Made Passive Flux Sampler Studies of Monoaromatic Hydrocarbons Emissions from Selected Commercially-Available Floor Coverings. *Build Environ* 2017, *123*, 1–13, doi:10.1016/j.buildenv.2017.06.035.
12. Zabiegała, B.; Sărbu, C.; Urbanowicz, M.; Namieśnik, J. A Comparative Study of the Performance of Passive Samplers. *J Air Waste Manage Assoc* 2011, *61*, 260–268, doi:10.3155/1047-3289.61.3.260.
13. Marć, M.; Zabiegała, B.; Namieśnik, J. Application of Passive Sampling Technique in Monitoring Research on Quality of Atmospheric Air in the Area of Tczew, Poland. *Int J Environ Anal Chem* 2014, *94*, 151–167, doi:10.1080/03067319.2013.791979.

Disclaimer/Publisher's Note: The statements, opinions and data contained in all publications are solely those of the individual author(s) and contributor(s) and not of MDPI and/or the editor(s). MDPI and/or the editor(s) disclaim responsibility for any injury to people or property resulting from any ideas, methods, instructions or products referred to in the content.
